# The inactive X chromosome drives sex differences in microglial inflammatory activity in human glioblastoma

**DOI:** 10.1101/2024.06.06.597433

**Published:** 2024-06-06

**Authors:** Marla E. Tharp, Claudia Z. Han, Chris D. Balak, Conor Fitzpatrick, Carolyn O’Connor, Sebastian Preissl, Justin Buchanan, Alexi Nott, Laure Escoubet, Konstantinos Mavrommatis, Mihir Gupta, Marc S. Schwartz, U Hoi Sang, Pamela S. Jones, Michael L. Levy, David D. Gonda, Sharona Ben-Haim, Joseph Ciacci, David Barba, Alexander Khalessi, Nicole G. Coufal, Clark C. Chen, Christopher K. Glass, David C. Page

**Affiliations:** 1. Whitehead Institute, Cambridge, MA 02142, USA.; 2. Department of Cellular and Molecular Medicine, University of California, San Diego, La Jolla, CA 92093, USA.; 3. Flow Cytometry Core Facility, The Salk Institute for Biological Studies, La Jolla, CA 92037, USA.; 4. Center for Epigenomics, University of California, San Diego, La Jolla, CA 92093, USA.; 5. Department of Brain Sciences, Imperial College London, London, United Kingdom.; 6. UK Dementia Research Institute, Imperial College London, London, United Kingdom.; 7. Bristol-Myers Squibb, San Diego, CA 92121, USA.; 8. Bristol-Myers Squibb, San Francisco, CA 94158 USA; 9. Department of Neurosurgery, University of California, San Diego, La Jolla, CA 92037, USA.; 10. Department of Neurosurgery, University of California, San Diego-Rady Children's Hospital, San Diego, CA 92123, USA.; 11. Department of Pediatrics University of California, San Diego, La Jolla, CA 92093, USA.; 12. Sanford Consortium for Regenerative Medicine, La Jolla, CA 92037, USA.; 13. Department of Biology, Massachusetts Institute of Technology, Cambridge, MA 02139, USA.; 14. Howard Hughes Medical Institute, Whitehead Institute, Cambridge, MA 02142, USA.; 15. Present address: Institute of Experimental and Clinical Pharmacology and Toxicology, Faculty of Medicine, University of Freiburg, Freiburg, Germany.; 16. Present address: Department of Neurosurgery, Yale University, New Haven, CT 06520, USA.; 17. Present address: Department of Neurosurgery, Massachusetts General Hospital, Harvard Medical School, Boston, MA 02114, USA.; 18. Present address: Department of Neurosurgery, University of Minnesota, Minneapolis, MN 55455, USA.; 19. These authors contributed equally.

## Abstract

Biological sex is an important risk factor in cancer, but the underlying cell types and mechanisms remain obscure. Since tumor development is regulated by the immune system, we hypothesize that sex-biased immune interactions underpin sex differences in cancer. The male-biased glioblastoma multiforme (GBM) is an aggressive and treatment-refractory tumor in urgent need of more innovative approaches, such as considering sex differences, to improve outcomes. GBM arises in the specialized brain immune environment dominated by microglia, so we explored sex differences in this immune cell type. We isolated adult human TAM-MGs (tumor-associated macrophages enriched for microglia) and control microglia and found sex-biased inflammatory signatures in GBM and lower-grade tumors associated with pro-tumorigenic activity in males and anti-tumorigenic activity in females. We demonstrated that genes expressed or modulated by the inactive X chromosome facilitate this bias. Together, our results implicate TAM-MGs, specifically their sex chromosomes, as drivers of male bias in GBM.

## Introduction:

For reasons not fully understood, cancers outside of the reproductive tract show higher incidence and mortality rates in men, while women generally display stronger immune responses that more effectively clear pathogens but increase susceptibility to autoimmune diseases^1,2^. Since the immune system plays a vital role in controlling tumor development, sex differences in tumor-immune interactions may drive sex-biased outcomes in cancer.

How tumor-immune interactions lead to sex differences in cancer is a complex problem and may depend on 1) the stage of tumor development and 2) the tissue in which the tumor resides. It is necessary to dissect and compare the male and female immune responses in these distinct tumor environments. In early stages of tumor development, immune cells have enhanced anti-tumorigenic properties and immune surveillance mechanisms to eliminate newly transformed tumor cells^3^. In later stages, however, tumor cells evolve the ability to evade immune attack and even promote a pro-tumorigenic immunosuppressive microenvironment^3^. Additionally, aspects of inflammation can have opposing and paradoxical effects on tumor progression. For example, pro-inflammatory activity can drive tumor cell killing and, at the same time, tumor-supportive angiogenesis and chronic inflammation if not efficiently resolved^3,4^.

The brain features a highly specialized immune system and dismal tumor prognoses, presenting an important setting in which to explore sex differences established by tumor immunity. Glioblastoma multiforme (GBM) is the most common and lethal primary brain tumor in adults, and among the most refractory to immunotherapy^5,6^. The brain environment contributes to poor prognoses in GBM by restricting circulating cytotoxic immune cells and relying on brain-resident macrophages called microglia for immune functions. GBM has a 1.6:1 male-biased incidence, with females showing about 10 months longer survival following diagnosis^5^. There is suggestive evidence that brain tumor immunity underlies the observed male-bias in GBM because, among four subtypes of GBM distinguished by gene expression and morphological features, three subtypes are male-biased, and these three display the highest infiltration of immune cells^7^. Given that microglia are the primary immune cell in the brain and heavily infiltrate GBM tumors, we hypothesize that sex differences in GBM are established by cell-autonomous genetic and molecular mechanisms in this highly enriched immune cell type^8^.

Little is known about genetic and molecular mechanisms regulating TAM-MG (tumor-associated macrophages enriched for microglia) phenotypes in human GBM, or how they might differ between males and females. Microglia display remarkable plasticity, constantly surveying their local environment and responding to brain insults, including tumors, by engaging specific sets of transcription factors to activate new gene expression programs that yield distinct phenotypic states^9,10^. TAM-MG phenotypes in GBM are diverse and complex due to the heterogeneous nature of the tumors, a consequence of dynamic shifts in proportions of infiltrating immune cells, multiple tumor cell lineages, and niches of necrosis, uncontrolled proliferation, and hypoxia^11^. Often, TAM-MGs display tumor-supportive and tumor-killing phenotypes in the same tumor, based on the local signals TAM-MGs encounter, leading to immunologically hot and cold regions of the tumor that are difficult to treat^12-14^. Understanding how biological sex factors into the regulation of different TAM-MG phenotypes, and the relationship to male-bias in GBM, will be a significant step toward more effective therapies.

Studies in mice suggest sex differences in brain tumor development and survival are mediated by TAM-MGs. For example, microglia-enriched expression of Junction Adhesion Molecule A (JAM-A) was found to regulate pathogenic immune activation exclusively in female tumors, leading to better survival outcomes in female mice^15^. In other mouse studies, brain tumor immune cell composition and gene expression, measured by single-cell RNA-seq, showed an increased pro-inflammatory signature and proportion of infiltrating monocytes in females versus males, while males showed increased expression of tumor-supportive genes and proportion of immunosuppressive macrophages^16^. However, rigorous investigations into sex differences in human TAM-MGs and the regulatory units underlying these differences have not been reported.

To investigate sex differences in human TAM-MGs and their role in establishing male bias in GBM, we generated and analyzed transcriptomic and epigenomic data from purified adult human TAM-MGs and control microglia. We found that, compared to males, female TAM-MGs exhibited stronger anti-tumorigenic inflammatory responses in low-grade gliomas, which may enhance tumor cell killing and slow tumor growth in less advanced tumor environments. In contrast, male TAM-MGs showed increased pro-tumorigenic proliferative and inflammatory activity in high-grade GBM, which may underlie worse GBM outcomes in males. We demonstrated that sex differences in TAM-MG inflammatory response activation and resolution are facilitated by genes expressed or modulated by the inactive X chromosome. Our studies demonstrate a pivotal role for TAM-MGs in establishing male-biased GBM incidence and mortality, and directly link the genetic underpinnings of this male bias to the sex chromosomes.

## Results:

### TAM-MG gene expression across tumor grades

**Figure 1.**
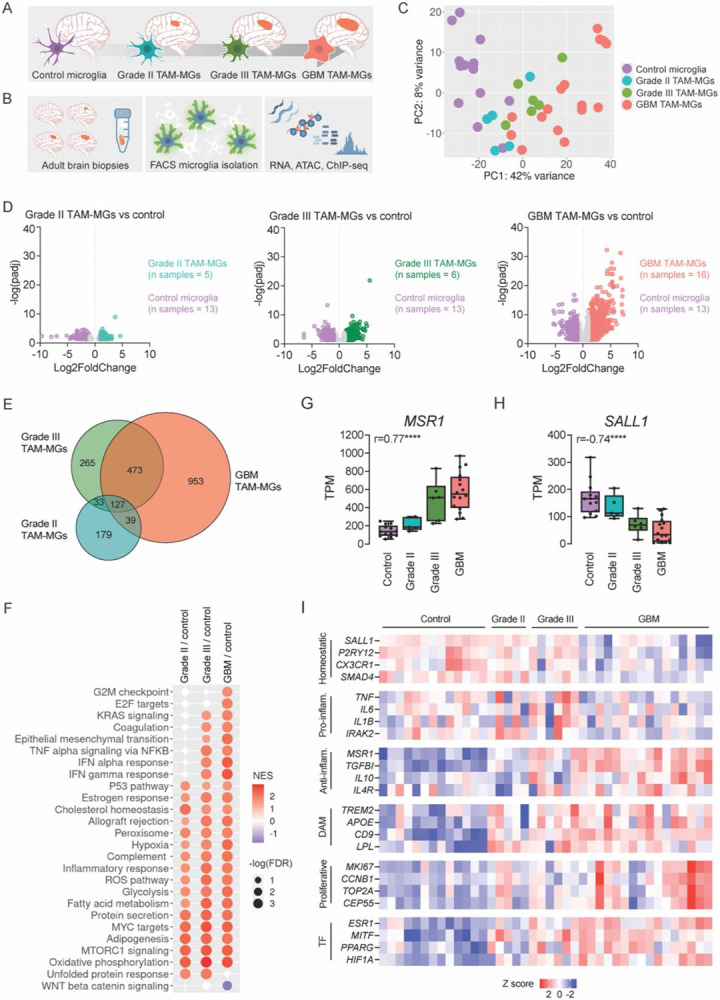
TAM-MG gene expression across tumor grades A. Project design overview: investigating genetic and epigenetic regulators of the human TAM-MG state using control microglia and TAM-MGs of grades II, III, and IV (GBM). B. Experimental design: microglia isolation from human brain tumor resections and control brain tissue by FACS, followed by transcriptomic and epigenomic assays. C. Principal component analysis (PCA) of bulk RNA-seq libraries from human TAM-MGs and control microglia. D. Volcano plots of differentially expressed genes in TAM-MGs vs control microglia across tumor grades. E. Venn diagram of overlapping and unique differentially expressed genes across tumor grades. F. GSEA analysis using Hallmark gene sets for grade II, III, and GBM TAM-MGs vs control microglia. G. *MSR1* gene expression positively correlates with tumor grade. H. *SALL1* gene expression negatively correlates with tumor grade. I. Genes representative of characterized microglia states whose expression correlates with tumor grade and that are differentially expressed in TAM-MGs compared to control microglia.

We generated transcriptomic and epigenomic data from human TAM-MGs isolated from adult, surgically resected tumors, including grade II and III gliomas and (grade IV) GBM (Fig. 1A, Table S1). As controls, we studied microglia isolated from brain biopsies of individuals undergoing surgery for epilepsy^17,18^. Brain tumors were graded based on genetic and morphological criteria defined by the World Health Organization^19^. Cells of low-grade tumors divide slowly and rarely spread beyond the central nervous system, while cells of high-grade tumors divide and spread more quickly. GBM is the highest-grade tumor and is distinguished from other gliomas by 1) wildtype isocitrate dehydrogenase *IDH* gene, 2) regions of necrosis, 3) excessive and aberrant neovascularization, and 4) increased macrophage infiltration^19,20^. TAM-MG and control microglia populations were stringently FACS sorted using expression of CD11b^+^, CD45^mid^, CX3CR1^mid^, CD64^+^, and CCR2^lo^ to exclude inflammatory macrophages and recently immigrated monocytes (Fig. 1B, Fig. S1A-D). Bulk RNA-seq, ATAC-seq, and H3K27ac ChIP-seq were performed on sorted TAM-MG and control microglia samples (Fig. 1B, Table S2). Upon performing principal component analysis on bulk RNA-seq transcriptomes, we observed that TAM-MG samples clustered primarily by tumor grade, and secondarily by other variables such as age, primary vs. recurrent tumor status, and *IDH* mutation status (Fig. 1C, Table S1).

We first identified differentially expressed genes (DEGs) between control microglia and TAM-MGs (Table S3). Since TAM-MGs from grade II, grade III, and GBM tumors formed separate clusters by principal component analysis, we found DEGs between control microglia and TAM-MGs for each grade individually (Fig. 1D). DEGs that distinguished control microglia from TAM-MGs of grade II and grade III tumors were similar, while DEGs that distinguished control microglia from GBM TAM-MGs were mostly unique, underscoring the substantial differences in the tumor microenvironment between gliomas and GBM (Fig. 1E).

We performed gene set enrichment analysis (GSEA) on the same control vs TAM-MG comparisons for each tumor grade to investigate how TAM-MG pathways were affected by these different tumor environments. Querying the fifty “Hallmark” gene sets^21^, we observed a subset of gene sets enriched in TAM-MGs from all tumor grades compared to control microglia, and another subset enriched only in TAM-MGs from high-grade and GBM tumors (Fig. 1F). Gene sets enriched in TAM-MGs from all grades included metabolic processes like adipogenesis, glycolysis, and oxidative phosphorylation, upregulation of which may be required for TAM-MG survival in the tumor microenvironment limited in nutrients and oxygen (Fig. 1F). In TAM-MGs from high-grade and GBM tumors, we observed greater enrichment of gene sets involved in cell proliferation and interferon signaling pathways (Fig. 1F). GBMs exhibit a substantially increased proliferation rate compared to gliomas, indicating an environment abundant in mitogens that may influence TAM-MGs. Additionally, glioma stem cells have been shown to increase the expression of *Csf1*, a key factor for microglia proliferation and survival^22^. Previous studies using single-cell analysis to map cellular populations in human GBM tumors also found specific populations of pro-inflammatory and proliferative microglia^9^. Together, our GSEA identified pathways that are enriched in all TAM-MGs and may be required for viability in the tumor microenvironment, as well as pathways that are enriched only in high grade and GBM TAM-MGs and may drive worse tumor outcomes.

To identify genes involved in the TAM-MG state transition, we calculated the correlation of each gene’s expression with tumor grade across TAM-MG and control microglia samples. One of the genes most positively correlated with tumor grade was the scavenger receptor *MSR1/CD204* (Fig. 1G). *MSR1* is a known TAM-MG biomarker, and its expression has been associated with decreased survival in GBM^23^. In contrast, *SALL1*, a critical microglia lineage-determining and homeostatic gene, was negatively correlated with tumor grade (Fig. 1H)^24^. The intersection of our correlation analysis with gene sets derived from microglia homeostatic and reactive states revealed additional microglia homeostatic genes whose expression declined with tumor grade, including *P2RY12* and *CX3CR1* (Fig. 1I). Genes of the disease-associated microglia (DAM) phenotype related to phagocytosis and lipid metabolism increased in expression with tumor grade in TAM-MGs, including *TREM2,* a receptor recognizing glycoproteins and lipids that is widely expressed in myeloid cells. Interestingly, TREM2 activation is often considered protective in the context of Alzheimer’s disease but deleterious in tumors through elicitation of an immunosuppressive environment (Fig. 1I)^25,26^. Genes related to proliferative and inflammatory microglia states also increased in TAM-MGs, confirming our GSEA (Fig. 1I). Interestingly, anti-inflammatory genes like *MSR1* and *IL10* showed a strong positive correlation to tumor grade, while pro-inflammatory genes like *IL-6* and *TNF* were more highly expressed in grade II-III gliomas (Fig. 1I). These inflammatory gene signatures highlight the complexity of TAM-MG immune phenotypes that may underlie worse tumor outcomes.

Finally, we observed genes encoding transcription factors like *MITF*, *PPARG*, *ESR1*, and *HIF1A* with significant positive expression correlation with tumor grade (Fig. 1I). MiTF/TFE transcription factors regulate genes involved in lysosomal function, autophagy and phagocytosis that are also increased in TAM-MGs^27^. *PPARG* and *ESR1* also have critical functions in cancer progression and interactions with sex hormones that may contribute to sex differences in TAM-MGs underlying GBM outcomes^28,29^.

### TAM-MG enhancer activation

Microglia phenotypes in disease are governed by differential transcription factor binding and enhancer activation in response to changes in local environmental cues^10^. Given the large number of differentially expressed genes in GBM TAM-MGs compared to control microglia and increased expression of transcription factors known to influence microglia state, we hypothesize that there may be significant changes in microglia enhancer activation upon association with GBM tumors. The chromatin landscape in TAM-MGs has been explored using single-cell ATAC-seq in human and mouse gliomas, which has the advantage of being able to separate subpopulations of immune cells from other tumor cells, but is limited by uneven sequencing coverage and transcript biases. Nonetheless, chromatin accessibility distinguishing TAM-MGs from other infiltrating immune cell populations^30^ and different TAM-MG phenotypic states across gliomas^31^ have been reported. However, the active enhancers and their interacting transcription factors regulating the human TAM-MG state have not been explored. For this, the addition of H3K27ac ChIP-seq is required, providing a surrogate for active promoters and enhancers, rather than open chromatin detected by ATAC-seq that can indicate repressed, poised, or active elements.

**Figure 2.**
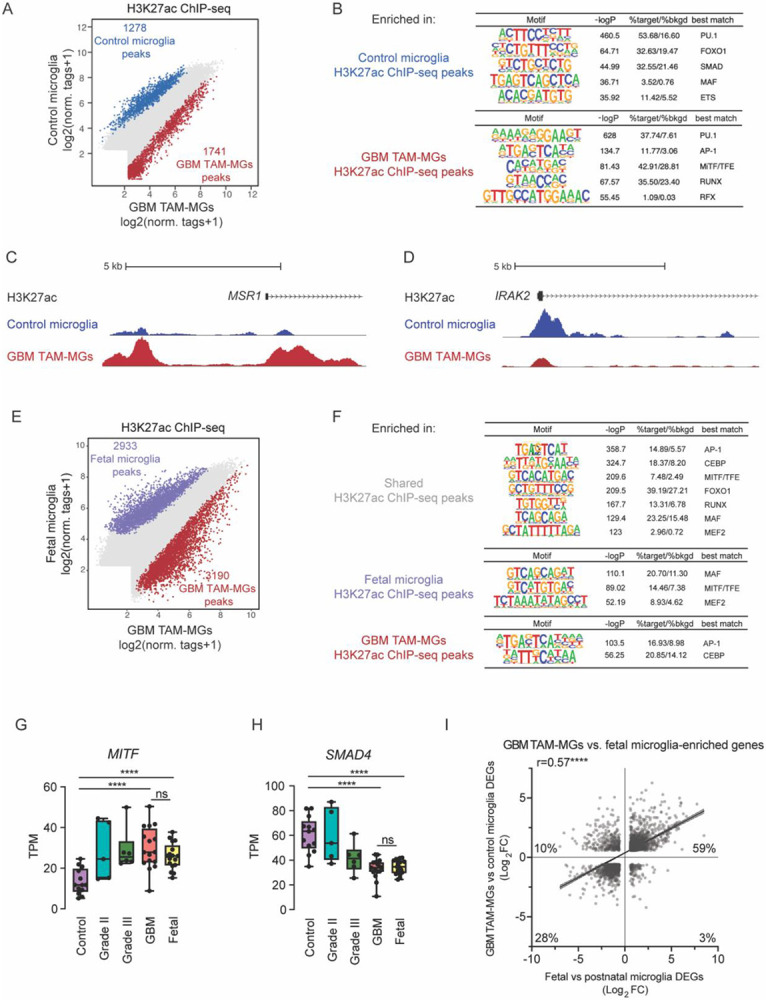
A. Differential H3K27ac ChIP-seq peaks (log2 (normalized tags +1)) in control microglia (blue) and GBM TAM-MGs (red). B. Transcription factor binding motifs enriched in H3K27ac ChIP-seq peaks from control microglia and from GBM TAM-MGs. C. UCSC browser tracks showing greater enhancer activity near *MSR1* in GBM TAM-MGs compared to control microglia by H3K27ac ChIP-seq. D. UCSC browser tracks showing greater enhancer activity near *IRAK2* in control microglia compared to GBM TAM-MGs by H3K27ac ChIP-seq. E. Differential H3K27ac ChIP-seq peaks (log2 (normalized tags +1)) in fetal microglia (blue), GBM TAM-MGs (red). F. Transcription factor binding motifs shared between, and enriched in, H3K27ac ChIP-seq peaks from fetal microglia and from GBM TAM-MGs. G. Expression of GBM TAM-MG and fetal microglia-enriched transcription factor *MITF*. H. Expression of control microglia and postnatal microglia-enriched transcription factor *SMAD4*. I. Comparison of differentially expressed genes common to GBM TAM-MGs vs control microglia and fetal microglia vs postnatal microglia.

We conducted a genome-wide assessment of active enhancers that differ between human TAM-MGs and control microglia. Comparison of active enhancer landscape between GBM TAM-MGs and control microglia revealed 1741 differentially active regions in GBM TAM-MGs, while 1278 peaks were differentially active in control microglia (Fig. 2A). Application of *de novo* motif analysis showed strong enrichment for motifs for transcription factor FOXO1 and SMAD family members in control microglia and motifs matching the MiTF-TFE and RFX family members in GBM TAM-MGs (Fig. 2B). Differentially active regions in GBM TAM-MGs and control microglia correspond to nearby differentially expressed genes. For example, H3K27ac signal in the promoter region for *MSR1* was greater in GBM TAM-MGs compared to control microglia, and for *IRAK2* was greater in control microglia compared to GBM TAM-MGs (Fig. 2C-D). This supports *MSR1* and *IRAK2* gene expression increasing and decreasing, respectively, between TAM-MGs and control microglia (Fig. 1H). MiTF-TFE factors are master regulators of lysosomal function, autophagy, and phagocytosis, while the RFX family have been implicated in cell cycle^32^, immune system maturation^33^, and cilial development^34^. SMAD4 interacts with SALL1 to promote microglia maturation during fetal brain development^24^. These results led us to compare GBM TAM-MG transcription factor families to those recently identified in human microglia along a developmental context^18^. SMAD and FOXO1 motifs enriched in control microglia compared to GBM TAM-MGs were also enriched in postnatal microglia compared to fetal microglia. MiTF-TFE and RFX motifs enriched in GBM TAM-MGs compared to control microglia were also enriched in fetal microglia compared to postnatal microglia^18^. To gain a better understanding of the similarities between the transcriptional networks of human fetal microglia and GBM TAM-MGs, we compared their active enhancer landscapes and performed motif analysis on active peaks that were shared (Fig. 2E). As expected, we found enrichment of MiTF-TFE motifs, as well as AP-1 and MAF motifs (Fig. 2F). Interestingly, when comparing motifs enriched in active peaks of fetal microglia compared to GBM TAM-MGs, we also saw enrichment for MiTF-TFE, MAF and MEF2A in fetal microglia, while in GBM TAM-MGs, AP-1 and CEBP motifs were enriched, suggesting that MiTF-TFE may be enriched in both states, but act with specific binding partners (Fig. 2F).

We next investigated how the expression levels of these key transcription factors change with tumor grade. *MITF* expression increases in TAM-MGs with tumor grade, and expression levels are similarly high in GBM TAM-MGs and fetal microglia (Fig. 2G). In contrast, *SMAD4* expression decreases in TAM-MGs with tumor grade and expression levels are similarly low in GBM TAM-MGs and fetal microglia (Fig. 2H). Further, we compared all differentially expressed genes between (1) TAM-MGs and control microglia and (2) fetal and postnatal microglia. We found a significant overlap in the identity and directionality of differentially expressed genes between the two comparisons (Fig. 2I). Over half of the total shared DEGs were upregulated in TAM-MGs and fetal microglia (Fig. 2I). Recently, induced pluripotent cell-derived microglia (iMGs) or iMG engrafted into humanized mice (xMGs) have been shown to recapitulate significant aspects of the fetal to postnatal microglia transition, with iMGs more alike fetal microglia and xMGs more similar to postnatal microglia^35^. Thus, we also compared the differentially expressed genes between TAM-MGs and control microglia to differentially expressed genes between iMGs and xMGs and observed the same trend in which TAM-MG genes more significantly overlapped with those iMGs. (Fig. S2). Collectively, these results suggest that the GBM tumor microenvironment influences human microglia to assume a more embryonic state that potentiates development as opposed to immune regulation.

### Sex differences in TAM-MGs

**Figure 3.**
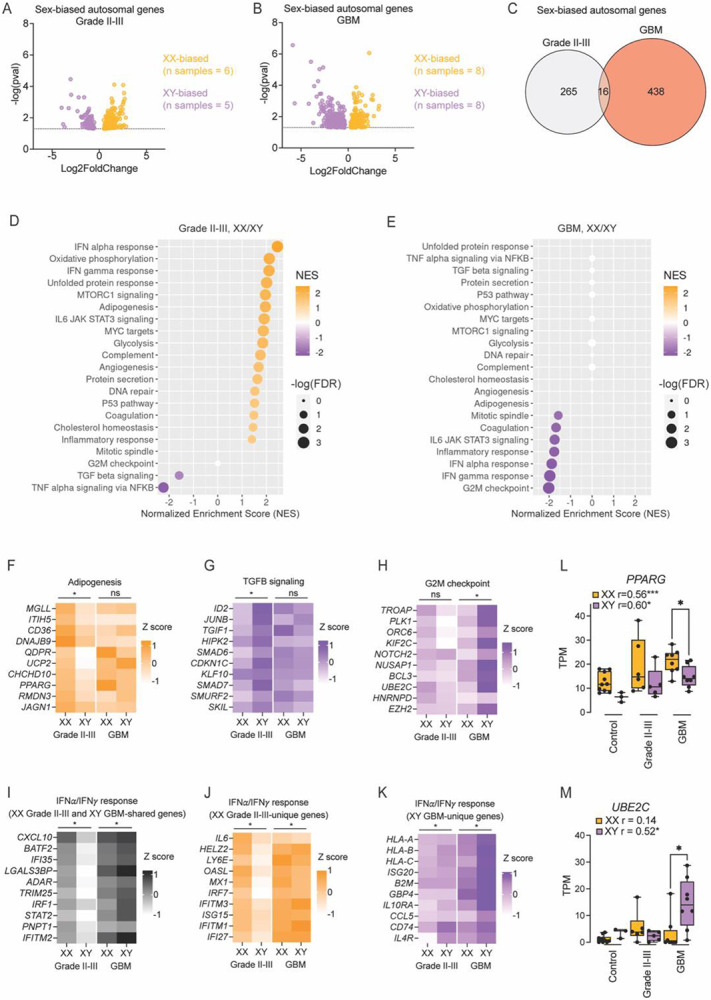
Sex differences in TAM-MGs A. Sex-biased autosomal genes in grade II-III TAM-MGs. B. Sex-biased autosomal genes in GBM TAM-MGs. C. Venn diagram of sex-biased genes in grade II-III TAM-MGs and GBM TAM-MGs. D. GSEA of sex-biased genes in grade II-III TAM-MGs considering Hallmark gene sets. E. GSEA of sex-biased genes in GBM TAM-MGs considering Hallmark gene sets. F. Top 10 leading-edge genes of XX-biased Adipogenesis gene set in grade II-III TAM-MGs. G. Top 10 leading-edge genes of XY-biased TGFB signaling gene set in grade II-III TAM-MGs. H. Top 10 leading-edge genes of XY-biased G2M checkpoint gene set in GBM TAM-MGs. I. Top 10 leading-edge genes of Interferon alpha and gamma response gene sets from both XX-biased grade II-III TAM-MGs and XY-biased GBM TAM-MGs. J. Top 10 leading-edge genes of Interferon alpha and gamma response gene sets from only XX-biased grade II-III TAM-MGs K. Top 10 leading-edge genes of Interferon alpha and gamma response gene sets from only XY-biased GBM TAM-MGs L. Representative XX-biased gene *PPARG*. M. Representative XY-biased gene *UBE2C*.

Given the male-biased GBM incidence and mortality rate and precedence for sex differences in immune regulation, we then asked if XX and XY TAM-MGs showed different responses in low-grade gliomas and high-grade GBM. We compared sex-biased gene expression in TAM-MGs from pooled grade II and grade III gliomas and TAM-MGs from grade IV GBM (Fig. 3A-B, Table S4A). We limited this analysis to autosomal genes since genes expressed from the Y chromosome and Xi will obscure global pictures of sex differences. We found that sex-biased autosomal genes in low-grade glioma mostly did not overlap with sex-biased genes in GBM, suggesting that sex-biased responses differ by tumor grade (Fig. 3C).

We performed GSEA to determine sex-biased pathways in TAM-MGs from the low-grade gliomas and high-grade GBM. Low-grade TAM-MGs showed XX-enrichment of pathways related to inflammatory activity, including the interferon alpha and interferon gamma response and IL6 JAK STAT3 signaling, as well as pathways involved in lipid metabolism, including adipogenesis and cholesterol homeostasis (Fig. 3D, F, I). XY-enriched gene sets were involved in TGF beta signaling and TNF alpha signaling via NFKB (Fig. 3D, G). IL6 and interferons are typically pro-inflammatory, anti-tumorigenic pathways, while TGF beta is immunosuppressive and pro-tumorigenic, suggesting that our results support worse outcomes in males in the low-grade glioma environment. Considering GBM TAM-MGs, no XX-enriched pathways were observed; however, XY TAM-MGs were enriched in proliferative and inflammatory pathways, including the interferon alpha and interferon gamma responses that were also observed to be XX-biased in low-grade gliomas (Fig. 3E, H, I). Given the vastly different environments of low-grade gliomas and GBM, we asked whether the genes driving the XX-biased interferon responses in low-grade glioma TAM-MGs were similar to those driving the XY-biased interferon responses in GBM TAM-MGs, to understand whether they are driving anti- or pro-tumorigenic effects. Genes that were unique to the XX-biased interferon responses in low-grade gliomas included *IL6* and *OASL* that mediate pro-inflammatory anti-tumorigenic activity (Fig. 3J). Upon GSEA using genes induced by known pro-inflammatory stimulus lipopolysaccharide (LPS) we found enrichment in XX TAM-MGs in low-grade gliomas, and no sex bias in GBM, further supporting the XX-biased inflammatory response being more anti-tumorigenic in low-grade gliomas (Fig. S3). Genes unique to XY-biased interferon responses in GBM included *IL4R* and *IL10RA* that mediate immunosuppressive pro-tumorigenic activity (Fig. 3K). Interferon response driver genes shared between these two different environments with opposing sex differences include *IRF1* and *STAT2*, and others that do not have clear pro- or anti-tumorigenic activities in TAM-MGs, but in the framework of male-bias in GBM, may be anti-tumorigenic in the low-grade glioma environment, but pro-tumorigenic in GBM. Together, the tumor grade-dependent sex differences in TAM-MG inflammatory responses may underlie male-biased incidence and mortality in GBM. The heightened ability for XX TAM-MGs to mount an acute pro-inflammatory response in less severe tumors may better suppress tumor growth early on in females, manifesting as male-biased incidence. Inflammatory and proliferative responses support tumor growth in the aggressive necrotic and angiogenic GBM microenvironment, and are XY-biased.

Next, we assessed sex-biased TAM-MG genes correlated with tumor grade, suggesting that they influence tumor progression. We compared the correlation of gene expression to tumor grade for each gene expressed in TAM-MGs and control microglia in male and female samples independently. Of the genes positively correlated with tumor grade, 37% were significant in both sexes, 42% significant only in XX samples, and 20% were significant only in XY samples. In the negative direction, 57% of genes were significantly correlated with tumor grade in both sexes, 27% significant only in XX samples, and 16% significant only in XY samples. Among the genes with sex-biased positive correlations with tumor grade, we found that *PPARG,* encoding a transcription factor involved in lipid metabolism and resolving inflammation, had a stronger correlation with tumor grade in XX compared to XY samples, and XX-biased expression in GBM TAM-MGs (Fig. 3L). Since XX TAM-MGs mount a stronger pro-inflammatory response in less advanced tumor environments, perhaps they also induce greater expression of machinery that resolves this response, such as *PPARG*, that is apparent in more advanced tumor environments like GBM. Genes showing XY-biased correlations with tumor grade included *UBE2C,* also male-biased in GBM TAM-MGs and involved in degrading cyclins for mitotic progression (Fig. 3M).

### Sex chromosome genetic drivers of sex differences in TAM-MGs

**Figure 4.**
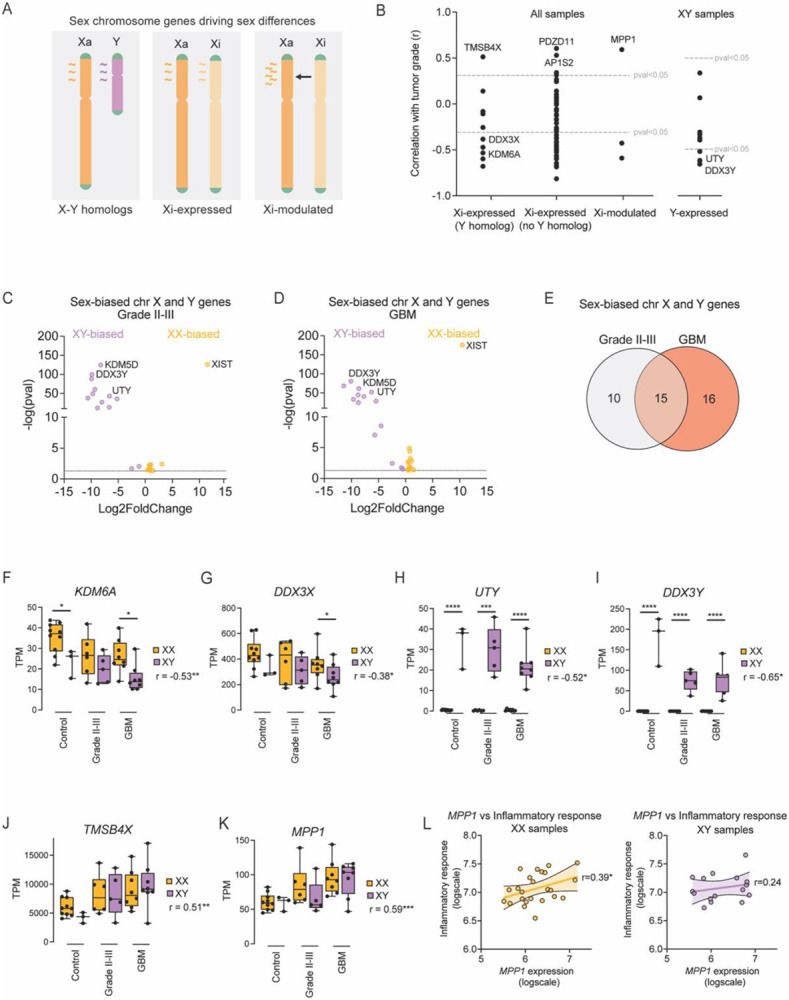
Sex chromosome genetic drivers of sex differences in TAM-MGs A. Model of sub-sets of sex chromosome genes with potential to drive sex differences. B. The correlation coefficient of sex chromosome gene expression with tumor grade, separated by sex chromosome gene sub-sets. Y-expressed gene correlations were only calculated in XY samples. C. Differentially expressed sex chromosome genes between XX and XY grade II-III TAM-MGs. D. Differentially expressed sex chromosome genes between XX and XY GBM TAM-MGs. E. Overlap of sex-biased genes in grade II-III TAM-MGs and GBM TAM-MGs. F. Expression of *KDM6A* in XX and XY TAM-MGs and control microglia. Significance of correlation and sex-bias indicated. G. Expression of *DDX3X* in XX and XY TAM-MGs and control microglia. Significance of correlation and sex-bias indicated. H. Expression of *UTY* in XX and XY TAM-MGs and control microglia. Significance of correlation and sex-bias indicated. I. Expression of *DDX3Y* in XX and XY TAM-MGs and control microglia. Significance of correlation and sex-bias indicated. J. Expression of *TMSB4X* in XX and XY TAM-MGs and control microglia. Significance of correlation indicated. K. Expression of *MPP1* in XX and XY TAM-MGs and control microglia. Significance of correlation indicated. L. Correlation of *MPP1* and the average expression of genes from the Inflammatory response gene set in XX and XY samples.

The sex chromosomes are the biological foundation of sex differences. We hypothesized that the sex chromosomes house putative drivers of sex-biased gene expression observed in TAM-MGs. Subsets of sex chromosome genes are likely candidates to regulate sex-biased phenotypes based on their evolution and dosage sensitivities (Fig. 4A). First, the X and Y chromosomes retain a set of dosage-sensitive homologous gene pairs throughout sex chromosome evolution that are broadly expressed across the body^36,37^. These X-Y pair genes often diverge in sequence due to the absence of genetic recombination between the X and Y chromosomes, leading to sex-biased expression and function. Second, the X chromosome comes in two epigenetically distinct forms, active (Xa) and inactive (Xi), to correct for dosage differences between XX and XY individuals. However, in humans, Xi maintains the expression of about one-third of its genes, although at reduced levels, leading to increased expression in females compared to males^38^. X chromosome genes retaining Y homologs are typically permitted to have the highest expression from Xi due to the presence of the Y partner. Last, Xi can express genes that modulate other X chromosome genes exclusively expressed from Xa, leading to increased expression in females^38^.

To determine whether sex chromosome genes served as genetic drivers of sex-biased expression patterns in TAM-MGs, we first found sex chromosome genes that changed in expression with tumor grade, and thus, may influence tumor progression (Table S4B). Considering the subsets of sex chromosome genes with the greatest potential to drive sex-biased phenotypes – Xi-expressed (with and without Y homologs), Xi-modulated, and Y-expressed genes – we found a correlation value for the expression of each gene with tumor grade (Fig. 4B). The first pattern that emerged was a significant negative correlation between many Xi and Y-expressed genes with tumor grade. A few Xi-expressed and one Xi-modulated gene had a significant positive correlation with tumor grade.

To better understand the potential for these tumor grade-responsive sex chromosome genes to drive sex differences, we quantified the sex-biased expression of X and Y chromosome genes in TAM-MGs from grades II-III tumors and TAM-MGs from GBM tumors. As expected, exclusive expression of Y chromosome genes was observed in XY TAM-MGs, and XX-biased expression of many genes expressed from Xi, both with and without Y homologs, was observed (Fig. 4C-D). Gene expression from the Y and Xi is largely conserved across cell types and disease states^39^, therefore, there was a larger proportion of overlapping sex-biased sex chromosome genes than sex-biased autosomal genes between grade II-III and GBM TAM-MGs (Fig. 4E, Fig. 3C).

Among the sex-biased genes negatively correlated with tumor grade were Xi-expressed and female-biased *KDM6A* and *DDX3X*, as well as their male-biased Y homologs *UTY* and *DDX3Y* (Fig. 4F-I). *KDM6A* and *DDX3X* are both implicated in inflammation and tumor immunity making them good candidate drivers of sex-biased inflammatory responses in TAM-MGs. Although combined expression of X and Y homologs for *KDM6A/UTY* and *DDX3X/DDX3Y* eliminates their sex-biased expression (Fig. S4A-B), functional differences between proteins encoded by X and Y homologs have been reported and can lead to sex-biased immune responses^40,41^. For example, the propensity to phase separate is stronger for DDX3Y than DDX3X under stressed conditions, resulting in DDX3Y driving stress granule assembly while DDX3X drives inflammasome assembly^40^.

There were no sex chromosome genes with both significant sex-biased expression and a significant positive correlation with tumor grade. However, the small set of positively correlated genes, including the Xi-expressed *TMSB4X* and Xi-modulated gene *MPP1*, had a strong positive correlation with tumor grade (Fig. 4J-K). *TMSB4X* and *MPP1* are also part of the transcriptional program upregulated in both TAM-MGs compared to control microglia and in fetal compared to postnatal microglia (Fig. S4C-D), and have known functions in cell adhesion, chemoattraction, and migration that are critical for tumor progression, as well as microglia-specific regulatory activity compared to other brain cell types (Fig. S4E-H)^42^. Interestingly, *MPP1* expression was significantly correlated with the average expression of genes from the Inflammatory response gene set in XX samples, but not XY (Fig. 4L). Therefore, although *MPP1* expression is not significantly sex-biased in our data, we hypothesize that it may be involved in regulating the sex-biased inflammatory response indirectly.

### Regulation of the inflammatory response by the Xi-expressed and modulated genes

**Figure 5.**
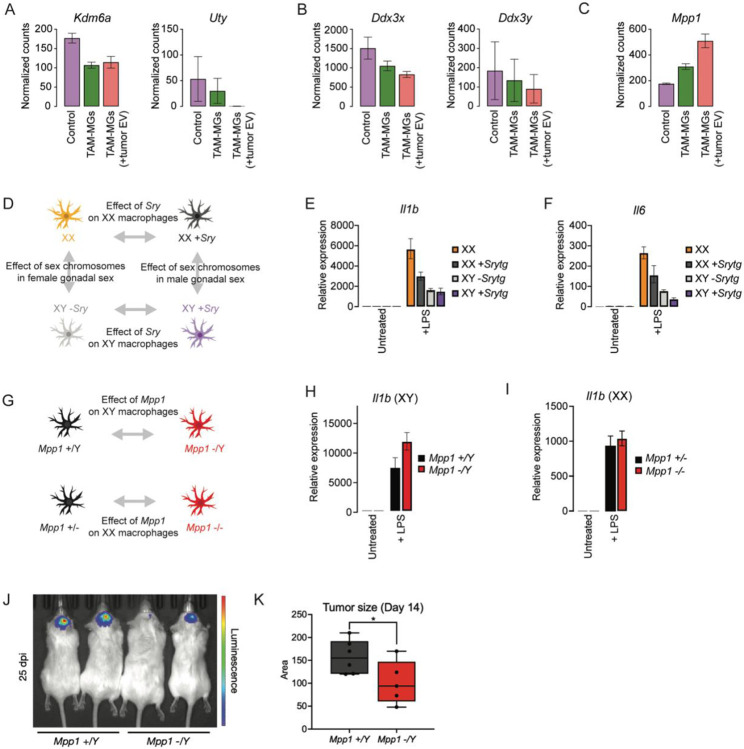
Regulation of the inflammatory response by the Xi-expressed and modulated genes A. Expression of Xi-expressed *Kdm6a* and Y homolog *Uty* downregulated in mouse TAM-MGs. B. Expression of Xi-expressed *Ddx3x* and Y homolog *Ddx3y* downregulated in mouse TAM-MGs. C. Expression of Xi-modulated *Mpp1* upregulated in mouse TAM-MGs. D. Schematic of sex-reversed mouse BMDM model. E. Expression of *Il1b* in XX, XX *+Sry*, XY −*Sry*, and XY +*Sry* BMDMs in untreated and LPS-treated conditions. F. Expression of *Il6* in XX, XX +*Sry*, XY −*Sry*, and XY +*Sry* BMDMs in untreated and LPS-treated conditions. G. Schematic of XX and XY *Mpp1* KO mouse BMDM model. H. Expression of *Il1b* in XY *Mpp1* knockout and wild-type BMDMs in untreated and LPS-treated conditions. I. Expression of *Il1b* in XX *Mpp1* knockout and wild-type BMDMs in untreated and LPS-treated conditions. J. Representative images of tumor development after 25 days in male *Mpp1* +*/Y* and *Mpp1 −/Y* mice. K. Quantification of tumor area after 14 days in male *Mpp1 +/Y* and *Mpp1 −/Y* mice.

We proceeded to test the role of the tumor grade-dependent, Xi-expressed or modulated genes of interest in the inflammatory response. Unlike the human Xi that maintains the expression of about one-third of its genes, only ~3% of genes are expressed from Xi in mice. However, the conserved set of Xi-expressed genes in humans and mice includes *Kdm6a*, *Kdm5c*, *Ddx3x*, and *Usp9x*, all of which are negatively correlated with tumor grade in human TAM-MGs. Interestingly, in a mouse model that tests the effects of tumor cell interactions on TAM-MG gene expression, analogous to human TAM-MG gene expression changes with enhanced tumor interactions that accompany increasing tumor grade, *Kdm6a* and *Ddx3x* also show decreased expression with tumor cell interactions (Fig. 5A-B)^43^. *MPP1*, the candidate Xi-modulated gene with a significant positive correlation to tumor grade in human TAM-MGs, also increased with tumor interaction in mouse TAM-MGs (Fig. 5C).

Since inflammation was a prominent sex-biased phenotype, being XX-biased in lower-grade tumors and XY-biased in GBM, we first tested the role of Xi-expressed genes in the pro-inflammatory response to the stimulus lipopolysaccharide (LPS). We chose LPS because sex-biased GSEA in our human TAM-MGs using a gene set of LPS-induced genes revealed similar sex-biased patterns to the inflammatory Hallmark gene sets. For example, female TAM-MGs showed enrichment of LPS-induced genes in low-grade tumors. However, no sex-biased enrichment of LPS-induced genes was observed in GBM, and given that LPS-induced genes are pro-inflammatory, this agrees with our observation that pro-inflammatory genes are reduced in GBM (Fig. S3).

We utilized a mouse macrophage system that can separate the effects of sex chromosomes from the effects of the male-determining gene *Sry* as a proxy for gonadal sex hormones through the insertion of a *Sry* transgene in an XX background and the deletion of *Sry* from an XY background^44,45^. In this way, we can compare how Xi-expressed genes (e.g. XaXi vs XaY −*Sry*), as well as gonadal sex hormones (e.g. XaXi vs XaXi +*Sry* and XaY +*Sry* vs XaY −*Sry*), impact the strength of LPS response (Fig. 5D).

We isolated bone marrow-derived macrophages (BMDMs) from the four genotypes and treated them with LPS for 24 hours. We quantified the induction of pro-inflammatory genes *Il-1b* and *Il-6* upon LPS stimulation by quantitative RT-PCR. In all conditions, LPS treatment significantly increased *Il-1b* and *Il-6* expression (Fig. 5E-F). However, *Il-1b* and *Il-6* induction was strongest in XX wild-type and XX +*Sry* BMDMs compared to XY −*Sry* and XY +*Sry* BMDMs (Fig. 5E-F). These results support a role for Xi in mounting a stronger pro-inflammatory response to LPS. This could explain the female-biased inflammatory response in less advanced tumor grades, but not in GBM tumors, as Xi-expressed genes decrease in abundance.

We then asked whether *MPP1* regulates the LPS response in XX and XY BMDMs (Fig. 5G). We stimulated *Mpp1* knockout BMDMs with LPS, and interestingly, observed sex-biased phenotypes. In XY BMDMs, the induction of *Il-1b* was significantly stronger in *Mpp1* knockout compared to wild-type, supporting its role in suppressing the LPS response in males (Fig. 5H). In contrast, in XX BMDMs, there was not a significant difference in the induction of *Il-1b* between *Mpp1* knockout compared to *Mpp1* heterozygous controls. Therefore, we conclude that the immunosuppressive function of *Mpp1* is male-biased (Fig. 5I). We validated this result using XY human stem cell-derived microglia with a CRISPRi knockdown of *MPP1* treated with LPS (Fig. S5A). *MPP1* knockdown was efficient at the RNA and protein levels and did not impede differentiation (Fig. S5B-C). *IL-1B* and *IL-6* induction were significantly increased in *MPP1* knockdown compared to non-targeting control knockdown XY stem cell-derived microglia (Fig. S5D-F).

To determine whether the immunosuppressive role of *Mpp1* in males influences tumor progression, we injected CT-2A glioma cells into *Mpp1* knockout and wildtype male mice. *Mpp1 −Y* mice developed smaller tumors than *Mpp1 +/Y* mice, suggesting that the immunosuppressive role of *Mpp1* in males drives tumor growth (Fig. 5J-K). Since *Mpp1* did not show immunosuppressive effects in females, *Mpp1* in TAM-MGs may mediate worse tumor outcomes in males. Such sex-biased tumor immunoregulatory phenotypes resemble those of *JAM-A*, which suppresses pathological microglia activation in females only, leading to larger brain tumors in female *JAM-A* knockout mice and better outcomes for females compared to males in wildtype conditions^15^.

Overall, we show that Xi-expressed genes drive a stronger response to LPS, while the Xi-modulated gene *MPP1* suppresses it in males only. We conclude that Xi-expressed genes conserved in humans and mice, like *DDX3X* and *KDM6A,* may drive female-biased inflammatory activity in low-grade tumors, given that females express these genes at higher levels and males express Y homologs that do not function as efficiently. Expression of *KDM6A* and *DDX3X* decreases in TAM-MGs with tumor grade, similar to other pro-inflammatory genes with known anti-tumorigenic roles, supporting these Xi-expressed genes in driving better GBM outcomes in females. Further, we conclude that sex-biased immunoregulatory functions of *MPP1*, which increases in abundance with tumor grade in both sexes, establishes additional female protection in GBM.

## Discussion:

Considering that GBM is a male-biased cancer and females typically mount stronger immune responses, we asked how TAM-MG interactions may establish sex differences in GBM. We analyzed sex differences in TAM-MGs in low-grade gliomas and GBM and found sex-biased inflammatory gene expression in both tumor environments. Specifically, female TAM-MGs showed enrichment of anti-tumorigenic, pro-inflammatory genes in lower-grade gliomas, while male TAM-MGs showed enrichment of pro-tumorigenic, anti-inflammatory genes in GBM. We characterized genetic drivers on the X chromosome that are expressed and modulated by the Xi in activating and resolving the inflammatory response, the mechanistic basis for our observed sex differences, and we demonstrated their roles using human and mouse microglia models. Our findings correlate Xi-driven pro-inflammatory activity with female-biased anti-tumorigenic inflammatory activity in TAM-MGs as an explanation for male bias in GBM.

### A model of sex-biased TAM-MG inflammatory response dynamics with GBM progression

By drawing on our collective findings, the effects of inflammation on tumor development, and male bias in GBM, we propose the following model that accounts for sex differences in TAM-MG inflammatory responses and GBM progression (Fig. S6).

We first delineated the homeostatic to TAM-MG transcriptomic landscape in both sexes and found that TAM-MGs from lower-grade gliomas retain more features of mature microglia and anti-tumorigenic activity than TAM-MGs from GBM. This may be due to low-grade TAM-MGs being less phenotypically altered by tumor cells, resulting in their heightened immune competence to mount an acute pro-inflammatory response for tumor cell killing. With increased tumor cell interactions and tumor progression, TAM-MG immune competence becomes dampened to permit immune evasion. For example, we observed more pro-inflammatory gene expression in TAM-MGs from grade II-III gliomas compared to GBM TAM-MGs, which expressed higher levels of anti-inflammatory genes and additional pro-tumorigenic pathways such as those involved in cell proliferation.

We then investigated sex differences in TAM-MGs from low-grade glioma and GBM environments. First, we found female-biased pathways that included a number of metabolic pathways, and inflammatory pathways driven by pro-inflammatory genes. These results support a female-biased anti-tumorigenic response in low-grade gliomas, an environment resembling early tumor development, and may ultimately manifest as male-biased incidence of tumors like GBM. We observed male-biased pathways in TAM-MGs from GBM, including proliferative and inflammatory pathways driven by immunosuppressive genes. These represent pro-tumorigenic pathways that may drive more aggressive GBM in males leading to male-biased mortality. In previous studies, subsets of pro-inflammatory microglia have also been associated with high-grade GBM and may reflect chronic inflammation that promotes tumor growth^12^. Increased expression of the anti-inflammatory factor *PPAPRG* in XX GBM TAM-MGs may be essential for suppressing the pro-tumorigenic chronic inflammation in GBM, given that XX TAM-MGs mount a stronger pro-inflammatory response in less advanced tumor environments or early in tumor development.

### Xi genes drive sex differences in TAM-MGs

We discovered genetic drivers from the X chromosome, specifically, genes expressed and modulated by the Xi, that account for sex-biased inflammatory responses in TAM-MGs. We have previously quantified Xi-expressed and modulated genes in both in vitro cultured fibroblasts and LCLs, as well as in vivo isolated CD4+ T cells and monocytes^38,39^. Our study is the first to demonstrate the role of specific Xi-expressed and modulated genes from these studies in the activation and resolution of inflammation experimentally.

#### 1) Xi-expressed genes promote a heightened pro-inflammatory response

In mouse macrophages treated with LPS, we observed XX karyotypes induce greater levels of genes encoding pro-inflammatory cytokines like *Il1b* and *Il6*. We found that this is likely independent of the sex hormone environment, as XX macrophages with an *Sry* transgene also mounted a stronger pro-inflammatory response than XY macrophages. We can narrow down the specific Xi-expressed genes underlying this response by conserved Xi expression in mouse and humans. *DDX3X* and *KDM6A* were the only female-biased Xi-expressed genes in human TAM-MGs with conserved TAM-MG and Xi expression patterns in mice. Although these genes have Y homologs, *DDX3Y* and *UTY*, these homologs have known functional differences from their X counterparts. For example, *DDX3X* and *DDX3Y* diverge in the 5’UTR, which upon stress, results in the preferential sequestration of *DDX3Y* into stress granules rather than participating in inflammasome activity like *DDX3X*^40^. *KDM6A* is a lysine demethylase that removes transcriptional repressive marks from histones. However, the Y-homolog *UTY* has reduced demethylase activity^41^. We speculate that the functionally divergent Y homologs and female-biased expression of X homologs of these X-Y pairs may be drivers of sex differences in TAM-MGs influencing inflammatory activity and GBM progression.

#### 2) Xi-modulated gene MPP1 has sex-biased immunoregulatory effects

The Xi-modulated gene *MPP1* is among the most positively correlated X chromosome genes with tumor grade in human TAM-MGs and control microglia, as well as in mouse TAM-MGs. *MPP1* is associated with several human diseases, including developmental abnormalities, heart failure, and acute myeloid leukemia ^46-48^. *MPP1* is involved in organizing plasma membrane lipid domains and signaling complexes^49^, and has a known role in immune cell polarization and chemoattraction^50^. Prior to our study, a role for *MPP1* in sex differences in disease was not explored. First, we observed a stronger correlation between *MPP1* and the average expression of Hallmark inflammatory genes in female TAM-MG and microglia samples compared to male, as well as between *MPP1* and *PPARG*, and anti-tumor gene involved in resolving inflammation. (Fig. S7A). We asked whether *MPP1* regulates the expression of *PPARG*, and whether this relationship contributes to sex-biased *Mpp1* inflammatory phenotypes observed in mouse BMDMs. We tested whether *Mpp1* regulates *Pparg* expression in both untreated and LPS-treated conditions using *Mpp1* knockout vs. wildtype BMDMs (Fig. S7B-C). Indeed, *Pparg* expression decreases in *Mpp1* knockout BMDMs compared to wild-type. *PPARG* is a well-known factor in resolving inflammation and anti-tumor effects that now may be regulated by *MPP1,* and given the female-biased expression of *PPARG* in GBM-TAMs, may be an interesting mechanism of *Mpp1*-mediated sex differences^28,51,52^.

### Female sex hormones may enhance the pro-inflammatory response in TAM-MGs

Although our study focused on the genetic drivers of sex differences from the sex chromosomes, we also observed gene expression patterns in TAM-MGs and phenotypes in our LPS experiments that support the influence of sex hormone signaling. First, estrogen receptor *ESR1* was significantly expressed in TAM-MGs and positively correlated with tumor grade. Effects of estrogen have been tested in models of GBM and report tumor suppressive effects that may contribute to male bias in the disease, but no study has investigated the role of *ESR1* or effects of estrogen specifically in TAM-MGs. In our experiment using macrophages from the sex-reversed mouse model, we found that XX macrophages treated with LPS mounted a stronger pro-inflammatory response than XY macrophages. However, we also found that wild-type XX macrophages mounted a stronger pro-inflammatory response than XX macrophages containing an *Sry* transgene, a proxy for male sex hormones, suggesting that female sex hormones also contribute to the stronger pro-inflammatory response. Additionally, we sorted TAM-MGs from CT-2A tumors and microglia from control brain tissue in *Mpp1* knockout and wildtype mice and found that *Esr1* is regulated by *Mpp1* in both cases (Fig. 8A-B). Importantly, *Esr1* increases in expression between control microglia and TAM-MGs in wildtype mice, similar to humans. We speculate that genes expressed and modulated by the Xi establish sex differences in TAM-MGs, but these effects may be further enhanced by interactions with estrogen signaling.

### Limitations of the study

GBM heterogeneity makes purification of microglia a challenge. Although using our FACS-gating strategy (CD11b^+^, CD45^mid^, CX3CR1^mid^, CD64^+^, and CCR2^low^) to isolate TAM-MGs, we observed negligible expression of monocyte marker *CCR2*, B-cell marker *CD38*, and neutrophil marker *S100A8* in GBM TAM-MGs, significant expression of *ITGA4/CD49*, a marker of monocyte-derived macrophages, in GBM TAM-MGs was observed (Fig. S1B). Monocyte-derived macrophages that occupy the GBM environment long enough will begin to express markers similar to microglia, resulting in their isolation with other TAM-MGs. Importantly, *ITGA4/CD49* expression was not sex-biased in GBM TAM-MGs, and thus, does not affect our sex differences analysis. Antibodies against ITGA4/CD49 have be used to increase the purity of FACS-isolated TAM-MGs^30^, but these were not yet published at the time of our sample preparation. We will test these in future follow-up studies involving human TAM-MG sorting.

## STAR Methods

### RESOURCE AVAILABILITY

#### Lead contact

Further information and requests for resources and reagents should be directed to and will be fulfilled by the lead contact, David. C. Page (dcpage@wi.mit.edu).

#### Materials availability

This study did not generate new unique reagents.

#### Data and code availability

- Raw RNA-Seq, ATAC-Seq, and ChIP-Seq data has been deposited to dpGAP and processed data has been deposited at github. Both are publicly available as of the date of publication. Accession numbers and DOIs are listed in the key resources table.
- Original code has been deposited at github and is publicly available as of the date of publication. The accession number is listed in the key resources table.
- Any additional information required to reanalyze the data reported in this paper is available from the lead contact upon request.

### EXPERIMENTAL MODEL AND STUDY PARTICIPANT DETAILS

#### Human tissue

Isolation of microglia was performed as previously described from brain tissue in excess of that required for diagnosis of pathology. For control microglia samples, all patients were undergoing surgery for epilepsy and epileptogenic focus resections. Surgeries were performed at Rady Children’s Hospital or through the UC San Diego Health (Jacobs Medical Center or UC San Diego Medical Center Hillcrest). All Tumor tissue resections were performed at UC San Diego Hospital. Adult patient consent was obtained for all brain tissue and was approved under a protocol by the UC San Diego and Rady Children’s Hospital Institutional Review Board (IRB 160531, IRB 171361). Brain tissue resections were transferred to the laboratory on ice and microglia isolation was immediately performed within three hours after resection. Patient charts were reviewed prior to surgery to confirm pathological diagnosis, medications, demographics, and timing of stereoelectroencephalography (SEEG). This study was performed in accordance with ethical and legal guidelines of the University of California institutional review board. Cell viability and sequencing libraries reported in this study met technical quality control standards and no other criteria were used to exclude samples. We complied with all relevant ethical regulations.

#### Mice

Male mice with an *Sry* deletion and carrying an *Sry* transgene on chromosome 3 (*Sry*^*tm;tg*^) were bred to wild-type female mice from the same crosses in the 129S4/SvJae genetic background to generate the four core genotypes of the sex-reverse model. Mice carrying the *Mpp1* targeted mutation C.129(B6)-*Mpp1*^*tm1Ahc*^/J and backcrossed at least 20 generation into the BALB/cJ genetic background were acquired from the Jackson Laboratory. The mice used in this study were bred and maintained at the Whitehead Institute. All animals were maintained, and procedures performed in accordance with the guidelines of the Massachusetts Institue of Technology (MIT) Division of Comparative Medicine, which is overseen by MIT’s Institutional Animal Care and Use Committee (IACUC). The animal care program at MIT/Whitehead Institute is accredited by the Association for Assessment and Accreditation of Laboratory Animal Care, International (AAALAC), and meets or exceeds the standards of AAALAC as detailed in the Guide for the Care and Use of Laboratory Animals. The MIT IACUC approved this research (no. 230-4000-510).

### METHOD DETAILS

#### Human microglia isolation

Dissection of human brain tissues was done manually into 2-3 mm pieces. Tissue pieces were immersed in homogenization buffer (HBSS (Life Technologies, 14175-095), 1% bovine serum albumin (Sigma-Aldrich, A3059), 1 mM EDTA)) and mechanically dissociated using a 2 ml polytetrafluoroethylene pestle (Wheaton, 358026). Brain homogenate was pelleted, filtered through 40 ⎧m filter, re-suspended in 37% isotonic Percoll (Sigma, P4937) and centrifuged at 600xg for 30 min at 16-18°C with minimal acceleration and no deceleration. Percoll enrichment was performed and pelleted cells were collected. Red blood cells were lysed (eBioscience, 00-4333-57). Remaining cells were washed twice with homogenization buffer, filtered with a 40 μm strainer (BD Falcon 352350). Incubation with Fc-receptor blocking antibody (Human TruStain FcX, BioLegend 422302) in homogenization buffer for 20 minutes on ice was performed. For FACS purification, cells were stained for 30 minutes on ice with the following cell surface marker antibodies at 1:100 dilution (BioLegend): CD11b-PE (301306, clone ICRF44,), CD45-APC/Cy7 (304014, clone HI30), CD64-APC (305014, clone 10.1), CX3CR1-PerCP/Cy5.5 (341614, clone 2A9-1), CD14-AF 488 (301811, clone M5E2), HLA-DR-PE/Cy7 (307616, clone L243), and CD192-BV510 (357217, clone K036C2). Viable cells were first gated using Zombie Violet (Biolegend, 423113) or DAPI and added just prior to sorting (1 μg/ml final concentration). A BD Influx (100-μm nozzle, 22 PSI, 2-drop purity mode, sample chilling) or BD FACS AriaFusion (100-μm nozzle, 20 PSI, Purity mode (a 1-2 drop sort mode), sample chilling) were used to sort microglia defined as live/DAPI^−^/Zombie violet^−^; CD11b^+^; CD45^Low^; CD64^+^; CX3CR1^High^; CD192-BV510^Low^ single cells. FlowJo software (Tree Star) was used to analyze FACS data.

#### mRNA isolation

##### Human microglia

Microglia post-FACS sorting were stored in TRIzol LS. Phenol-chloroform extraction was used to isolate total RNA from homogenates and stored at − 80°C until cDNA libraries preparation for RNA-seq.

##### Human iMGs and mouse BMDMs

500,000 cells per sample were washed with PBS and isolated using the RNeasy Plus Mini Kit (Qiagen) following the manufacturer’s instructions. RNA quality control was performed using the 5200 Fragment Analyzer System (Agilent).

#### Bulk RNA-seq

##### Human microglia

We prepared RNA-seq libraries as previously described^17^. mRNAs were incubated with Oligo d(T) Magnetic Beads (New England BioLabs S1419) and fragmented in 2x Superscript III first-strand buffer (ThermoFisher Scientific 18080051) with 10mM DTT (ThermoFisher Scientific 18080044) at 94°C for 9 minutes. Fragment mRNA was incubated with 0.5 μl of Random primers (3 mg/mL) (ThermoFisher Scientific 48190011), 0.5 μl of 50mM Oligo dT primer, (ThermoFisher Scientific, 18418020), 0.5 μl of SUPERase-In (ThermoFisher Scientific AM2694), 1 μl of dNTPs (10 mM) at 50°C for one minute. Then, 1 μl of 10mM DTT, 6 μl of H_2_O+0.02%Tween-20 (Sigma), 0.1 μl Actinomycin D (2 mg/mL), and 0.5 μl of Superscript III (ThermoFisher Scientific) were added to the mixture. Synthesis of cDNA was performed by incubating the resulting mixture in a PCR machine with the following program: 25°C for 10 minutes, 50°C for 50 minutes, and a 4°C hold. RNAClean XP beads (Beckman Coulter A63987) were used to purify the product according to manufacturer’s instructions and eluted with 10 μl of nuclease-free H_2_O. Resulting elution was then incubated with 1.5 μl of Blue Buffer (Enzymatics P7050L), 1.1 μl of dUTP mix (10 mM dATP, dCTP, dGTP and 20 mM dUTP), 0.2 mL of RNase H (5 U/mL Y9220L), 1.2 μl of H_2_O+0.02%Tween-20, and 1 μl of DNA polymerase I (Enzymatics P7050L) at 16°C overnight. Purification of DNA was executed using 3 μl of SpeedBeads (Thermo Fisher Scientific 651520505025) resuspended in 28 μl of 20% PEG8000/2.5M NaCl to final of 13% PEG. Elution of DNA with 40 mL nuclease free H_2_O+0.02%Tween-20 was performed and underwent end repair by blunting, A-tailing and adaptor ligation as previously described^54^ using barcoded adapters. PCR amplification of libraries was carried out for 12-15 cycles and a 200-500 bp product size selected by gel extraction. 51 cycles of sequencing was performed on a HiSeq 4000 (Illumina) or a NextSeq 500 (Illumina).

##### Human iMGs and mouse BMDMs

iMG RNA sequencing libraries were prepared using the Kapa Hyper mRNA Library Preparation Kit. BMDM RNA sequencing libraries were prepared using the IDT xGen Std RNA Library Preparation Kit. Paired-end 50x50 bp sequencing was performed on a NovaSeq 6000 (Illumina).

#### Assay for Transposase-Accessible Chromatin sequencing (ATAC-Seq)

Human microglia (30,000-50,000) were lysed in 50 μl lysis buffer (10 mM Tris-HCl pH 7.5, 10 mM NaCl, 3 mM MgCl_2_, 0.1% IGEPAL, CA-630, in water). Nuclei that resulted were centrifuged at 500 rcf for 10 minutes. Pelleted nuclei were resuspended in 50 μl transposase reaction mix (1x Tagment DNA buffer (Illumina 15027866), 2.5 μl Tagment DNA enzyme I (Illumina 15027865), and incubated at 37°C for 30 min on a heat block. Microglia were directly placed in 50 μl transposase reaction mix for isolations resulting in under 30,000 microglia and incubated for 37°C for 30 min. Zymo ChIP DNA concentrator columns (Zymo Research D5205) were used to purify DNA, followed by elution with 11 μl of elution buffer, and amplification using NEBNext High-Fidelity 2x PCR MasterMix (New England BioLabs M0541) with the Nextera primer Ad1 (1.25 μM) and a unique Ad2.n barcoding primer (1.25 μM) for 8-12 cycles. Size-selection of libraries was performed by gel excision for fragments that were 175-255 bp. Single-end sequencing was performed for 51 cycles on a HiSeq 4000 or NextSeq 500.

#### Chromatin immunoprecipitation-sequencing (ChIP-Seq)

FACS-sorted microglia were centrifugated at 300 rcf and resuspended in 1% PFA. Microglia were rocked for 10 minutes at room temperature. Quenching of PFA was performed using 2.625M glycine at 1 to 20 volume for 10 minutes at room temperature. Fixed microglia were washed two times and centrifuged at 800-1000 rcf for 5 minutes. Pellets were snap frozen in liquid nitrogen. Microglia snap-frozen pellets containing 250,000 to 500,000 cells were thawed on ice and resuspended using 130 μl of LB3 buffer (10 mM TrisHCl pH 7.5, 100 mM NaCl, 1 mM EDTA, 0.5 mM EGTA, 0.1% Na-Deoxycholate, 0.5% N-Lauroylsarcosine, 1x protease inhibitors). Microglia were transferred to AFA Fiber microtubes (Covaris, MA). Sonication was performed using a Covaris E220 focused-ultrasonicator (Covaris, MA) for 12 cycles of 60 secs (Duty: 5, PIP: 140, Cycles: 200, AMP/Vel/Dwell: 0.0). Post-sonication, samples were transferred to an Eppendorf tube. Triton X-100 was added to the sample for a final concentration of 1%. Supernatant was spun at 21,000 rcf and the pellet discarded. 1% of the total volume was saved as DNA input control and stored at −20°C until library preparation. 25 μl of Protein A DynaBeads (Thermo Fisher Scientific 10002D) and 1 μl of H3K27ac antibody (Active Motif, 39085) were added to the supernatant for the immunoprecipitation. Samples were rotated at 4°C overnight. Dynabeads were washed 3 times with Wash Buffer 1 (20 mM Tris-HCl pH 7.4, 150 mM NaCl, 2 mM EDTA, 0.1% SDS, 1% Triton X-100), three times with Wash Buffer 3 (10 mM Tris-HCl pH, 250 mM LiCl, 1 mM EDTA, 1% Triton X100, 0.7% Na-Deoxycholate), three times with TET (10 mM Tris-HCl pH 8, 1 mM EDTA, 0.2% Tween20), once with TE-NaCl (10 mM Tris-HCl pH 8, 1 mM EDTA, 50 mM NaCl) and resuspended in 25 μl TT (10 mM Tris-HCl pH 8, 0.05% Tween20). Input samples were adjusted to 25 μl with TT. NEBNext Ultra II DNA Library Prep kit (New England BioLabs E7645) was used to prepare sample and input libraries according to manufacturer’s instructions. Samples and inputs were de-crosslinked (RNase A, Proteinase K, and 4.5 μl of 5M NaCl) and incubated overnight at 65°C. PCR-amplification of libraries was performed using NEBNext High Fidelity 2X PCR MasterMix (New England BioLabs M0541) for 14 cycles. Size selection of libraries was performed by gel excision of fragments that were 225 to 500 bp. Single-end sequencing of libraries for 51 cycles on a HiSeq 4000 or NextSeq 500 was performed.

#### CRISPRi

We used the top two guide sequences for *MPP1* from the human CRISPRi v2 gRNA library. We tested these guides for robust *MPP1* knockdown. We also used intragenic (IG) control guides mapping to a gene-poor region on Chr 2.

We cloned guides into the sgOpti vector. We digested the vector using FastDigest BsmBI (ThermoFisher) and dephosphorylated the ends with FastAP (ThermoFisher) for 30 min at 37°C. We gel-purified the digested plasmid using the QIAquick Gel Extraction Kit (Qiagen). Prior to ligation, we phosphorylated and annealed each pair of oligos using T4 Polynucleotide kinase (New England BioLabs). We then ligated each insert into the backbone using Quick Ligase (New England BioLabs) for 10 min at room temperature, and transformed into NEB Stable Cells for amplification (New England BioLabs). We confirmed gRNA sequences by sequencing.

LentiX-293T cells (Takara) were cultured in DMEM with 2mM L-Glutamine, 100U/mL Penicillin-Streptomycin, and 10% FBS on plates coated with collagen (5 μg/cm^2^) to produce virus. For gRNA constructs, we plated 5x10^6^ LentiX-293T cells in one 10 cm plate the night before transfection. The next day, we co-transfected 4.5 μg of sgOpti, 4 μg of pCMV-VSV-G, and 6.5 μg of psPAX2 using TransIT-LT1 reagent (Mirus). We harvested virus-containing media once at 48 h and once at 72 h, pooled, and tested for successful viral production using Lenti-X GoStiX (Takara). We concentrated gRNA virus 10X using Lenti-X concentrator (Takara). We pooled virus-containing media across all plates and concentrated 100X.

We transduced an H1 human embryonic stem cell line expressing dCas9 constitutively and selected using 2 μg/mL puromycin (Sigma) beginning 24 h post infection. Cells were washed and fed daily with fresh StemFlex media containing puromycin. Stem cells remaining were passaged after three days and expanded or used for iMG differentiation. Puromycin treatment was stopped during iMG differentiation.

#### Human iMG differentiation

H1 ESCs were differentiated into iMGs according to Brownjohn et al., 2018. ESCs were grown on matrigel-coated 6-well plates in 3mL StemFlex media with 10X supplement and 100U/mL Penicillin-Streptomycin. ESCs were harvested using ReLeSR passaging reagent and cells collected by centrifugation at 850 rpm for 5 minutes. Cells resuspended in cold StemFlex media containing 10 μM Rock inhibitor, 50 ng/mL BMP4, 20 ng/mL SCF, and 50 ng/mL VEGF at a concentration of 10,000 cells / 100 μL. 100 μL added per well of a 96-well U-bottom plate. Plate centrifugated at 300G for 3 minutes and 4 °C. Half media replacement (50 μL) after 2 days. After 4 days, using wide-bore pipet tip, re-plate 10-16 embryoid bodies per well of a 6 well plate containing 4 mL per well of X-VIVO hematopoetic medium with 2 mM GlutaMax, 100U/mL Penicillin-Streptomycin, and 55 μM β-mercaptoethanol and 100 ng/mL M-CSF and 25 ng/mL IL-3. After 7 days, exchange media. After 14 days, collect supernatant containing macrophage precursors. Collect by centrifugation at 850 rpm for 5 minutes and resuspend in neurobasal media containing MCSF and IL-34. iMGs differentiated over the course of 7 days.

#### Mouse BMDM differentiation

Bones from all four limbs were cleaned and dissociated using mortar and pestle in a buffer of PBS with 1% penicillin/streptomycin and 1% glutamine. and bone marrow collected. Cells were strained through a 70⎧m pore strainer. Cells were collected by centrifugation at 500G for 5 minutes. Supernatant was removed and AKC lysis buffer added for 2 minutes on ice to remove red blood cells. Cells were collected by centrifugation and washed one more time in the PBS buffer. Cells resuspended in media containing 20ng/ml M-CSF to promote macrophage differentiation and 10% low IgG FBS and plated on plastic. Approximately 2 10 cm dishes per mouse. Wash off non-macrophages after 7 days and replace media. Use macrophages within the next few days for stimulation experiments.

#### LPS treatment

Cells were treated for 3 hours with 20 ng/ml LPS to stimulate a pro-inflammatory response. Cells were washed and collected for RNA isolation and quantitative RT-PCR or RNA-seq. For iMGs, five independent rounds of differentiation for each of the two *MPP1* and two control knockdowns were treated and used as technical replicates. Given that we only have one line with constitutive dCas9, we only used this line, but validated results in mouse BMDMs with an *Mpp1* targeted deletion.

#### Quantitative RT-PCR

500 ng RNA added to Superscript VILO cDNA synthesis reaction. cDNA diluted 1:3 and 3ul added to qRT-PCR reaction using 2X Power SYBR green qPCR master mix for total volume of 10 uL. Endogenous control genes *Actb* and *Gapdh* used. Three technical replicates quantified per sample.

#### Glioma mouse tumor model

A single cell suspension of 10,000 CT-2A-luciferase glioma cells in 3ul of PBS were injected using a Hamilton microliter syringe with a 26-gauge needle at 0.5ul/min by stereotactic injection into the left region of the brain parenchyma. The needle was left in place for 3 min, and withdrawn over 2 minutes. Mice were anesthetized using isoflurane inhalation and pain controlled using 1mg/kg buprenorphine and lidocaine at the injection site.

#### IVIS imaging

Glioma-bearing mice were injected with D-Luciferin at 150 mg/kg in PBS. Mice were anaesthetized with 2% isofluorane after 10 minutes, followed by luminescence imaging using the IVIS Spectrum In Vivo Imaging System.

#### Data analysis

##### Bulk RNA-seq

All human analyses were performed using human genome build hg38, and a custom version of the comprehensive GENCODE v24 transcriptome annotation described in San Roman et al., 2023. Reads were pseudoaligned to the transcriptome annotation, and expression levels of each transcript were estimated using kallisto software v0.42.5. Resulting count data were imported into R with the tximport package v1.14.0 for normalization using DESeq2 v1.26.0. Downstream analysis used only protein-coding genes (as annotated in ensembl v104) with exceptions described in San Roman et al., 2023. All mouse analyses were performed using mouse genome build mm10, and GENCODE vM15 transcriptome annotation. Reads were pseudoaligned and transcript counts estimated and normalized as described for human samples.

##### Differentially expressed genes

Differentially expressed genes between TAM-MGs and control microglia, and between XX and XY TAM-MGs for grade II-III and GBM, were found using DESeq2. For TAM-MG vs control microglia, differentially expressed genes log_2_FC > 0.58, adjusted-*p* <0.05. For sex-biased genes, we used a cutoff of log_2_FC > 0.58, *p* < 0.05. Venn diagrams generated using Whitehead Institute’s Bioinformatics and Research Computing tools.

##### Correlation analysis

The Pearson correlation coefficient between tumor grade and gene expression were calculated in Excel. Statistical significance of each correlation was determined by calculating the t value and then p-value < 0.05 in the positive or negative direction. TPM counts were used for gene expression in each of the 40 control microglia and TAM-MG samples. For grading, control microglia = 1, grade II TAM-MGs = 2, grade III TAM-MGs = 3 and GBM TAM-MGs = 4.

##### Gene set enrichment analysis

Gene set enrichment analysis was conducted using the GSEA verson 4.1.0 software and the 50 Hallmark pathways downloaded from the Molecular Signatures Database. Analysis was restricted to autosomal protein-coding and lincRNA genes, which were ranked by each gene’s t-statistic from the DESeq2 models for TAM-MG vs control or XX vs XY comparisons. Results were considered statistically significant if FDR < 0.05

##### ATAC-seq and ChIP-seq analysis

Peaks were called using HOMER’s findPeaks command with the following parameters: “-style factor - size 200 - minDist 200” for ATAC-seq experiments and “-style histone -size 500 -minDist 1000 - region” for ChIP-seq experiments. Peaks were merged with HOMER’s mergePeaks and annotated using HOMER’s annotatePeaks.pl using all tag directories. For ChIP-seq experiments, peaks were annotated around ATAC-seq peaks with the parameter “-size −500,500 -pc”. Subsequently, DESeq292 was used to identify the differentially chromatin accessible distal sites (1000bp away from known TSS) or proximal sites (<500bp away from known transcript) with p-adj < 0.05 and fold change > 2.

##### Motif analysis

De novo motif analysis was performed using HOMER’s findMotifsGenome.pl with either all peaks or random genome sequences as background peaks. Motif enrichment scoring was performed using binomial distribution under HOMER’s framework.

##### Image analysis

Tumor area based on luminescence signal was measured and quantified using ImageJ.

#### Statistical analyses

Various statistical tests were use to calucate p-values as indicated in the Methods Details, figure legends, or text. To calculate statistics and generate plots, we used R software, version 4.2.1. Gene expression differences were calculated with DESeq2 with Benjamini-Hochberg multiple testing correction. We considered results statistically significant when *p* < 0.05 or, when using multiple hypothesis correction, adjusted-*p* < 0.05 or FDR < 0.05.

#### Data Visualization

PCA and heatmaps were generated in R and other plots were made with ggplots2 in R with colors reflecting the scores/expression values, including z-scores, as noted in each figure. Browser images were generated from the UCSC Genome Browser.

## Supplementary Material

Supplement 1 **Supplemental Table 1.** TAM-MG and control microglia sample information.

Supplement 2 **Supplemental Table 2.** TAM-MG and control microglia expressed genes (TPM).

Supplement 3 **Supplemental Table 3.** TAM-MGs vs control microglia differentially expressed genes.

Supplement 4 **Supplemental Table 4.** Sex-biased genes in low-grade and GBM TAM-MGs.

Supplement 5

## Figures and Tables

**Table:** KEY RESOURCES TABLE

REAGENT or RESOURCE	SOURCE	IDENTIFIER
**Antibodies**		
Anti-human CD11b PE (clone ICRF44)	Biolegend	301306; RRID: AB_314158
Anti-human CD45 APC-Cy7 (clone HI30)	Biolegend	304014; RRID: AB_314402
Anti-human CD45 (clone HI30)	Biolegend	304001; RRID: AB_314389
Anti-human CD64 APC (clone: 10.1)	Biolegend	305014; RRID: AB_1595428
Anti-human CX3CR1 PerCP-Cy5.5 (clone: 2A9-1)	Biolegend	341614; RRID: AB_11219203
Anti-human CD14-AF 488 (clone M5E2)	Biolegend	301811; RRID: AB_493159
Anti-human HLA-DR PE-Cy7 (clone L243)	Biolegend	307616; RRID: AB_493588
Anti-human CX3CR1 (clone 2A9-1)	Biolegend	341602; RRID: AB_1595422
Anti-human CD192-BV510 (clone K036C2)	Biolegend	357217; RRID: AB_2566504
Rabbit anti-mouse/human OLIG2 A647 (clone EPR2673)	Abcam	ab225100; RRID: AB_10861310
Mouse anti-mouse/human NeuN AF488 (clone A60)	MilliporeSigma	MAB377X; RRID: AB_2149209
Rabbit anti-mouse/human PU.1 PE (clone 9G7)	Cell Signaling	81886S; RRID:AB_2799984
Mouse anti-H3K27ac (clone MABI 0309)	Active Motif	39085; RRID: AB_2793305
**Chemicals, Peptides and Recombinant Proteins**		
KAPA SYBR FAST qPCR Master mix (2X)	Kapa Biosystems	Cat#07959427001
Dynabeads Protein A	Thermo Fisher Scientific	Cat#10002D
SpeedBeads magnetic carboxylate modified particles	GE Healthcare	Cat#65152105050250
TRIzol LS Reagent	Thermo Fisher Scientific	Cat#10296028
Formaldehyde, 37% by weight	Thermo Fisher Scientific	Cat#F79-1
Dulbecco’s PBS (DPBS) solution	Thermo Fisher Scientific	Cat#MT21031CV
Disuccinimidyl glutarate (DSG)	ProteoChem	Cat#C1104
Dimethyl sulfoxide (DMSO)	MilliporeSigma	Cat#D2650
UltraPure DNase/RNase-free distilled water	Thermo Fisher Scientific	Cat#10977023
Glycine	MilliporeSigma	Cat#4810
1M Tris-HCl, pH 8.0	Thermo Fisher Scientific	Cat#15568025
0.5 M EDTA, pH 8.0	Thermo Fisher Scientific	Cat#15575020
1M MgCl_2_	Thermo Fisher Scientific	Cat#AM9530G
Sucrose	Thermo Fisher Scientific	Cat#S6500
Triton X-100	MilliporeSigma	Cat#T8787
1,4-Dithiothreitol	Thermo Fisher Scientific	Cat#BP172-5
Bovine serum albumin	MilliporeSigma	Cat#A3059
4′,6-Diamidino-2-phenylindole, dilactate (DAPI)	BioLegend	Cat#422801
Oligo d(T)_25_ Magnetic Beads	NEB	Cat#S1419S
DTT	Thermo Fisher Scientific	Cat#P2325
SUPERase-In	Ambion	Cat#AM2696
Oligo dT	Thermo Fisher Scientific	Cat#18418020
Random Primers	Thermo Fisher Scientific	Cat#48190011
Agencourt RNA Clean XP Beads	Beckman Coulter	Cat#A63987
10X Blue Buffer	Enzymatics	Cat#P7050L
DNA Polymerase I	Enzymatics	Cat#P7050L
SuperScript III Reverse Transcriptase	Thermo Fisher Scientific	Cat#18080044
5x First-strand Buffer	Thermo Fisher Scientific	Cat#18080044
Actinomycin D	Sigma	Cat#A1410
DMEM / F12 (1:1) (1X)	Thermo Fisher Scientific	Cat#11330-032
GlutaMAX (100X)	Thermo Fisher Scientific	Cat#35050-061
MEM NEAA (100X)	Thermo Fisher Scientific	Cat#11140-050
Penicillin-Streptomycin	Thermo Fisher Scientific	Cat#15140-122
B-27 Supplement (50X)	Thermo Fisher Scientific	Cat#17504-044
N-2 Supplement (100X)	Thermo Fisher Scientific	Cat#17502-048
Recombinant Human M-CSF	Peprotech	Cat#300-25
Recombinant Human IL-34	Peprotech	Cat#200-34
Recombinant Human IL-3	Peprotech	Cat#200-03
Recombinant Human BMP4	Peprotech	Cat#120-05
Recombinant Human SCF	Peprotech	Cat#300-07
Recombiniant Human VEGF 121	Peprotech	Cat#100-20A
StemFlex Medium	Thermo	Cat# A3349401
Matrigel Matrix hESC-qualified	Corning	Cat#354277
X-VIVO 15 serum-free media	Lonza	Cat#02-053Q
Mouse M-CSF	Peprotech	Cat 315-02
2-mercaptoethanol (50mM)	Thermo Fisher Scientific	Cat#31350010
ReLeSR	STEMCELL Technologies	Cat#05872
Y-27632	STEMCELL Technologies	Cat#72308
LPS from *Escherichia coli* O55:B5	Sigma	Cat#L2637
**Critical Commercial Assays**		
RNeasy Plus Micro Kit	Qiagen	Cat#74034
Superscript VILO cDNA synthesis	Thermo	Cat#11754050
Direct-zol RNA MicroPrep Kit	Zymo Research	Cat#R2062
Qubit dsDNA HS Kit	Thermo	Cat#Q32851
Nextera DNA Library Prep Kit	Illumina	Cat#FC-121-1030
NEBNext Ultra II DNA library prep kit	NEB	Cat#E7645L
ChIP DNA Clean and Concentrator Kit	Zymo Research	Cat#D5205
**Experimental models: Cell lines**		
H1 hESC	WiCell	WA01; RRID:CVCL_9771
CT-2A-luciferase mouse glioma	Sigma	Cat#SCC195
**Experimental models: Organisms/Strains**		
Mouse: C.129(B6)-*Mpp1^tm1Ahc^*/J	JAX	Strain #:034592 RRID:IMSR_JAX:034592
Mouse: Srytm1;tg	N/A	Wang et al., 2013^44^
**Data**		
Human fetal microglia RNA-, ATAC-, H3K27ac-ChIP-seq	N/A	Han et al., 2023^18^
Human postnatal microglia RNA-, ATAC-, H3K27ac-ChIP-seq	N/A	Han et al., 2023^18^ dbGaP: phs001373.v2.p2
Human TAM-MG RNA-, ATAC-, H3K27ac-ChIP-seq	N/A	This paper
iMG, xMG RNA-seq	N/A	Han et al., 2023^18^
*MPP1* knockdown iMGs RNA-seq	N/A	This paper
*Mpp1^−/Y^* vs WT BMDMs RNA-seq	N/A	This paper
**Software and Algorithms**		
Bowtie2	Langmead and Salzberg, 2012^53^	http://bowtie-bio.sourceforge.net/bowtie2/index.shtml
FlowJo	N/A	https://www.flowjo.com/
HOMER	Heinz et al., 2010^54^	http://homer.ucsd.edu/homer/
R package: Pheatmap	N/A	https://CRAN.R-project.org/package=pheatmap
R package: Tidyverse	N/A	https://CRAN.R-project.org/package=tidyverse
R package: RColorBrewer	N/A	https://CRAN.R-project.org/package=RColorBrewer
R package: MACS2	N/A	https://github.com/macs3-project/MACS
R package: chromVar	N/A	https://bioconductor.org/packages/release/bioc/html/chromVAR.html
DESeq2 v1.38.3	Love et al., 2014^55^	https://bioconductor.org/packages/release/bioc/html/DESeq2.html
Kallisto	Bray et al., 2016^56^	https://pachterlab.github.io/kallisto/
R v4.2.1	The R Foundation	https://www.r-project.org
Gene Set Enrichment Analysis v4.1.0	N/A	https://www.gsea-msigdb.org/gsea/index.jsp
GraphPad Prism	N/A	N/A
Illustrator	Adobe	https://www.adobe.com/products/illustrator.html
**Other**		
BD Influx	BD	Equipment
BD FACSARIA Fusion	BD	Equipment
MoFlo Astrios	Beckman Coulter	Equipment
QuBit 4 Fluorometer	ThermoFisher	Equipment
5200 Fragment Analyzer System	Agilent	Equipment
NovaSeq 6000	Illumina	Equipment

## Inclusion and diversity

We support inclusive, diverse, and equitable conduct of research.
