# Supplementary material for: The inactive X chromosome drives sex differences in microglial inflammatory activity in human glioblastoma
Source: bioRxiv. 2024 Jun 6:2024.06.06.597433. Preprint. [Version 1] doi: 10.1101/2024.06.06.597433 (PMC11185629; doi:10.1101/2024.06.06.597433)
Supplement: Supplementary file 1 [file NIHPP2024.06.06.597433v1-supplement-5.pdf]

bioRxiv preprint doi: https://doi.org/10.1101/2024.06.06.597433; this version posted June 6, 2024. The copyright holder for this preprint (which was not certified by peer review) is the author/funder, who has granted bioRxiv a license to display the preprint in perpetuity. It is made available under aCC-BY 4.0 International license.

Supplementary Information

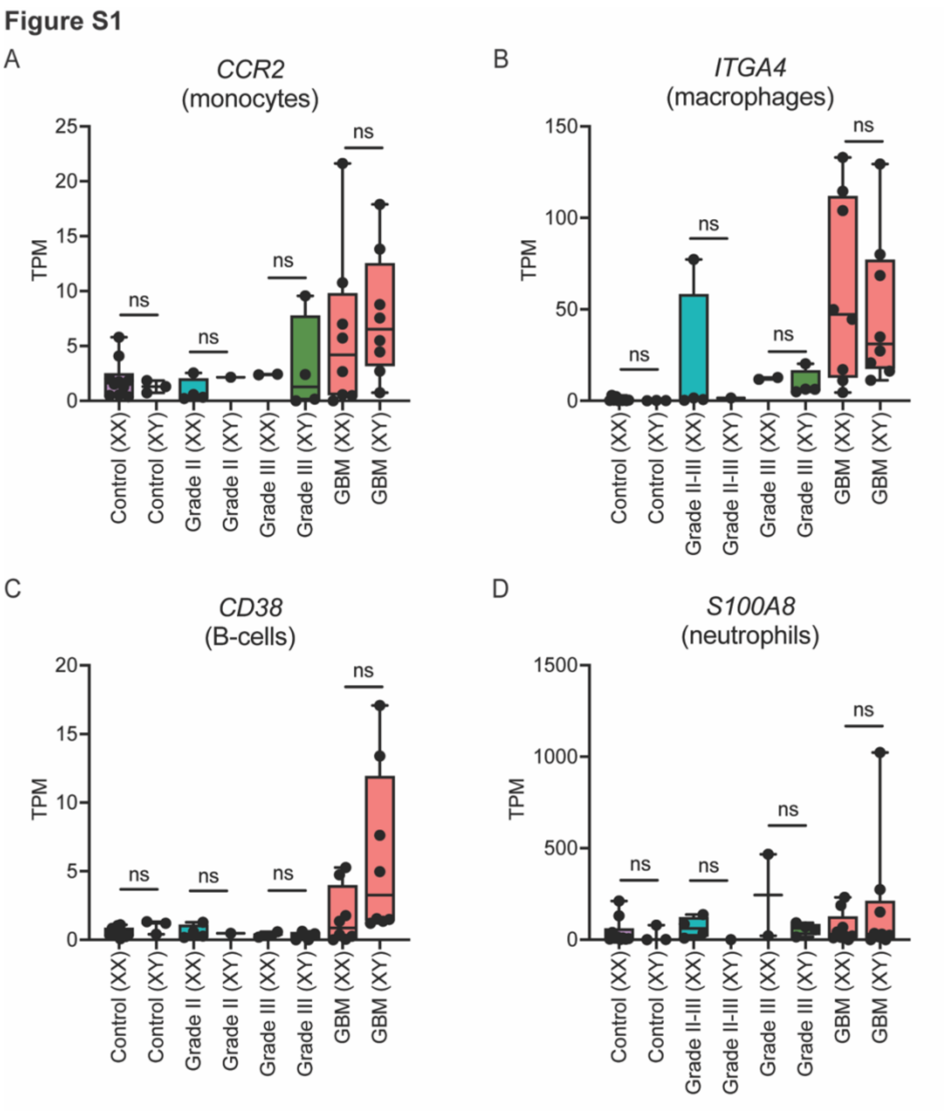

Supplemental Figure 1.

A. Expression of monocyte marker *CCR2* across control microglia and TAM-MGs, XX and XY.
B. Expression of blood-derived macrophage marker *ITGA4/C49* across control microglia and TAM-MGs, XX and XY.
C. Expression of B-cell marker *CD38* across control microglia and TAM-MGs, XX and XY.
D. Expression of neutrophil marker *S100A8* across control microglia and TAM-MGs, XX and XY.

bioRxiv preprint doi: https://doi.org/10.1101/2024.06.06.597433; this version posted June 6, 2024. The copyright holder for this preprint (which was not certified by peer review) is the author/funder, who has granted bioRxiv a license to display the preprint in perpetuity. It is made available under aCC-BY 4.0 International license.

Figure S2

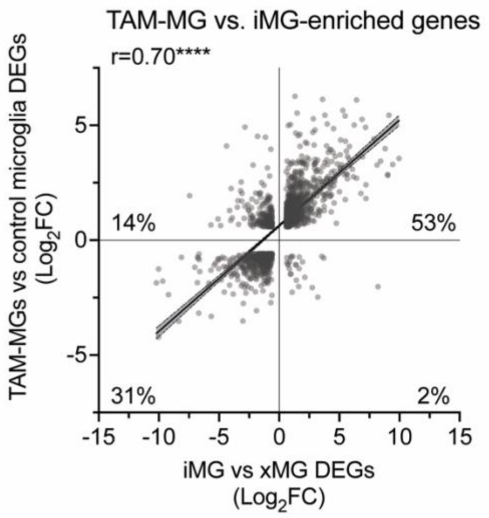

Supplemental Figure 2.

4-way comparison of differentially expressed genes common to TAM-MG vs control microglia and iPSC-derived microglia precursors vs iPSC-derived and xenografted microglia.

bioRxiv preprint doi: https://doi.org/10.1101/2024.06.06.597433; this version posted June 6, 2024. The copyright holder for this preprint (which was not certified by peer review) is the author/funder, who has granted bioRxiv a license to display the preprint in perpetuity. It is made available under aCC-BY 4.0 International license.

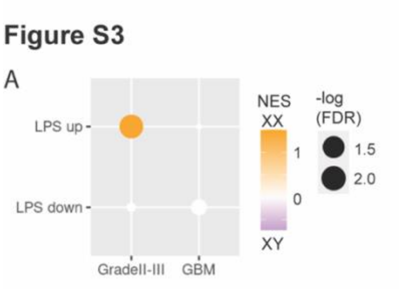

Supplemental Figure 3

GSEA for LPS response-upregulated and LPS response-downregulated genes between XX and XY TAM-MGs in grades II-III and GBM tumors.

bioRxiv preprint doi: https://doi.org/10.1101/2024.06.06.597433; this version posted June 6, 2024. The copyright holder for this preprint (which was not certified by peer review) is the author/funder, who has granted bioRxiv a license to display the preprint in perpetuity. It is made available under aCC-BY 4.0 International license.

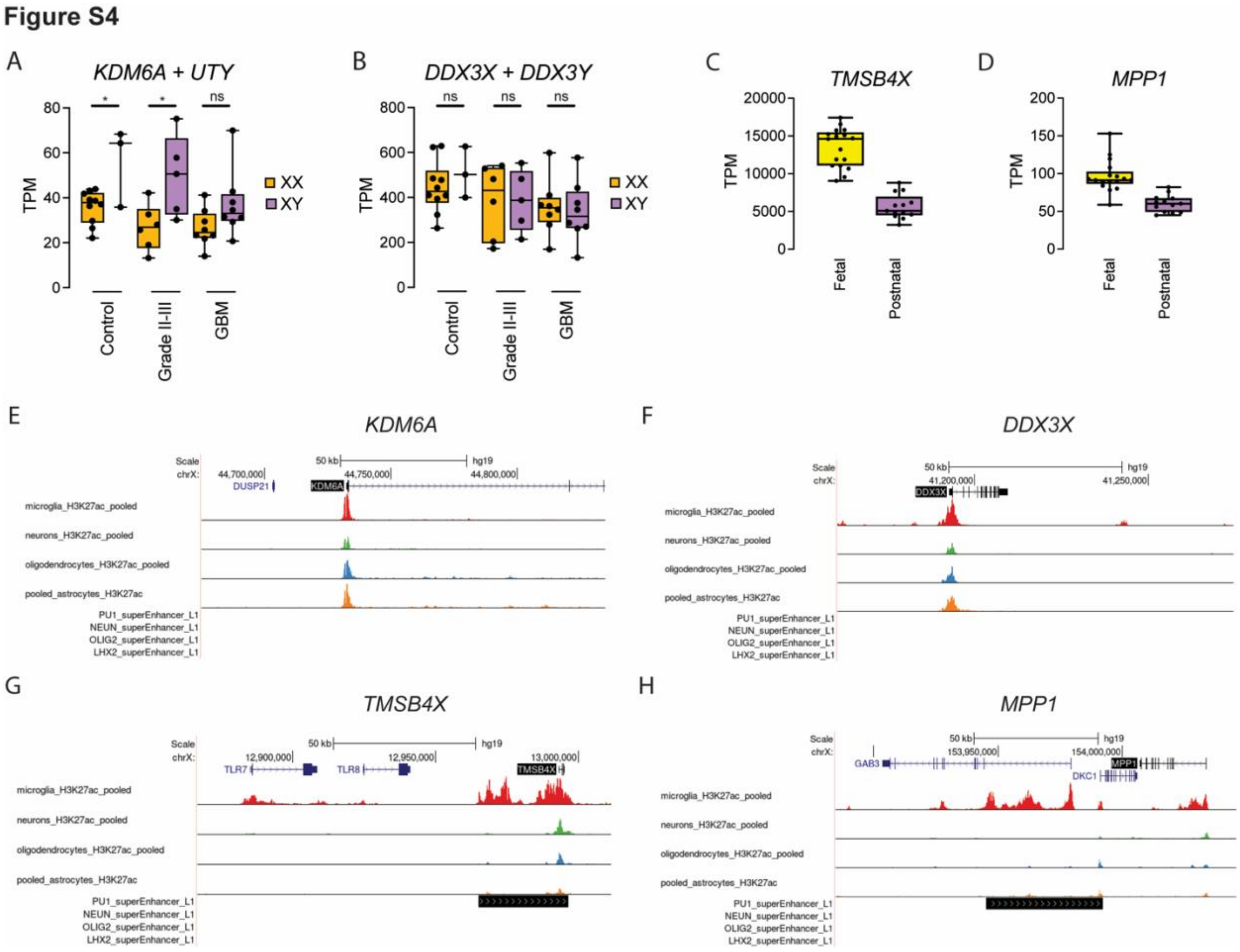

Supplemental Figure 4

A. Combined expression of *KDM6A/UTY* for each control microglia and TAM-MG sample.

B. Combined expression of *DDX3X/DDX3Y* for each control microglia and TAM-MG sample.

C. Expression of *TMSB4X* in human fetal and postnatal microglia.

D. Expression of *MPP1* in human fetal and postnatal microglia.

E. Active enhancers marked by H3K27ac ChIP-seq near *KDM6A* in human brain cell types.

F. Active enhancers marked by H3K27ac ChIP-seq near *DDX3X* in human brain cell types.

G. Active enhancers marked by H3K27ac ChIP-seq near *TMSB4X* in human brain cell types.

H. Active enhancers marked by H3K27ac ChIP-seq near *MPP1* in human brain cell types.

bioRxiv preprint doi: https://doi.org/10.1101/2024.06.06.597433; this version posted June 6, 2024. The copyright holder for this preprint (which was not certified by peer review) is the author/funder, who has granted bioRxiv a license to display the preprint in perpetuity. It is made available under aCC-BY 4.0 International license.

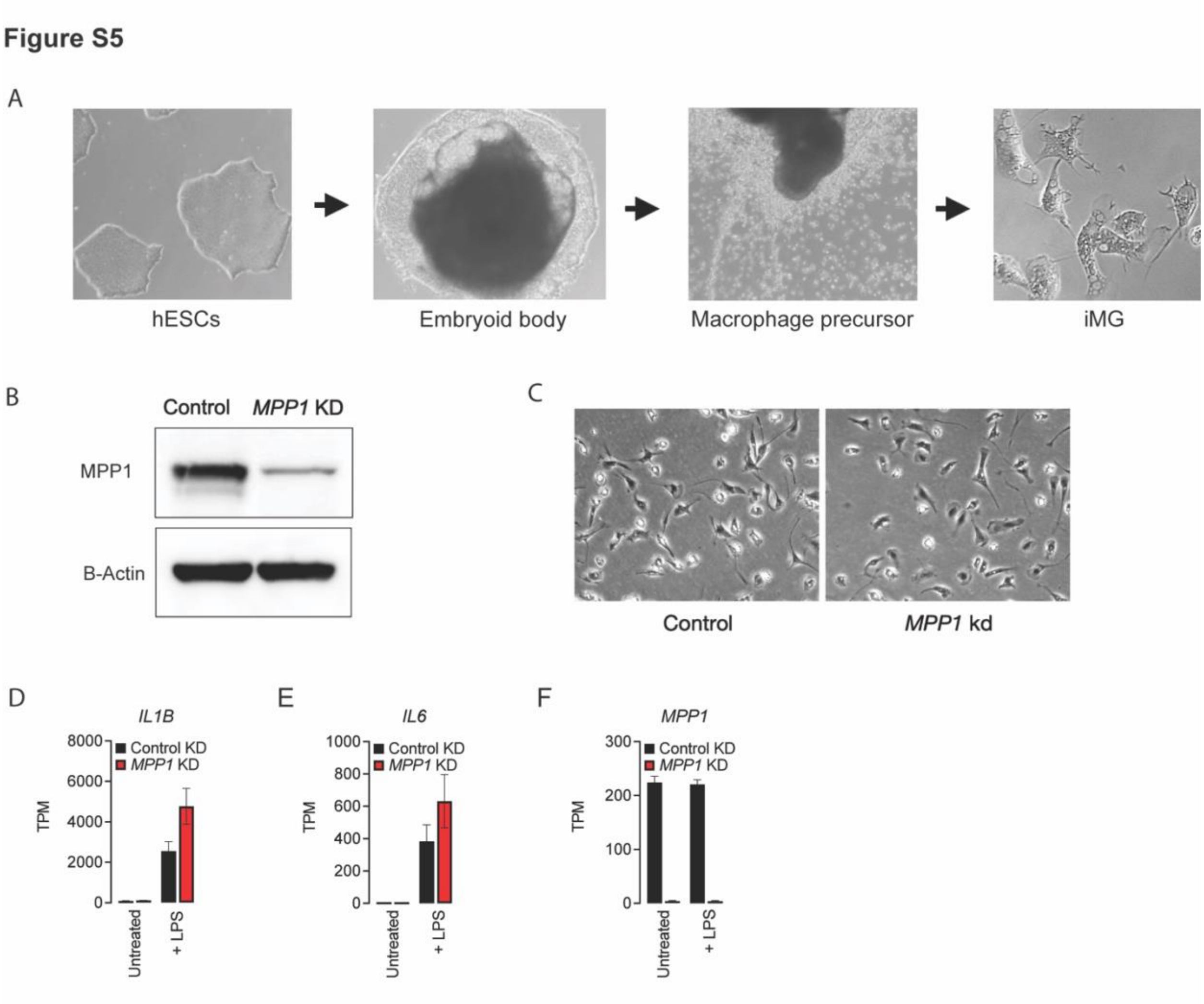

Supplemental Figure 5.

A. Method to differentiate human stem cell-derived microglia (iMGs).

B. MPP1 protein levels in control and *MPP1* knockdown iMGs.

C. Morphology of control and *MPP1* knockdown iMGs.

D. Expression of *IL1B* in control and *MPP1* knockdown iMGs, untreated and LPS-treated.

E. Expression of *IL6* in control and *MPP1* knockdown iMGs, untreated and LPS-treated.

F. Expression of *MPP1* in control and *MPP1* knockdown iMGs, untreated and LPS-treated.

bioRxiv preprint doi: https://doi.org/10.1101/2024.06.06.597433; this version posted June 6, 2024. The copyright holder for this preprint (which was not certified by peer review) is the author/funder, who has granted bioRxiv a license to display the preprint in perpetuity. It is made available under aCC-BY 4.0 International license.

Figure S6

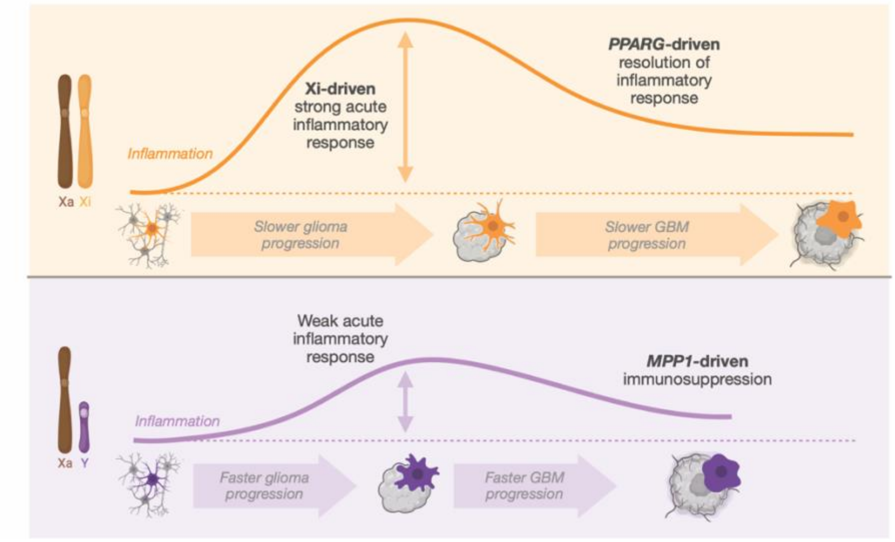

Supplemental Figure 6.

Model of sex differences in TAM-MGs inflammatory responses in low-grade gliomas and GBM and sex-biased genetic drivers.

bioRxiv preprint doi: https://doi.org/10.1101/2024.06.06.597433; this version posted June 6, 2024. The copyright holder for this preprint (which was not certified by peer review) is the author/funder, who has granted bioRxiv a license to display the preprint in perpetuity. It is made available under aCC-BY 4.0 International license.

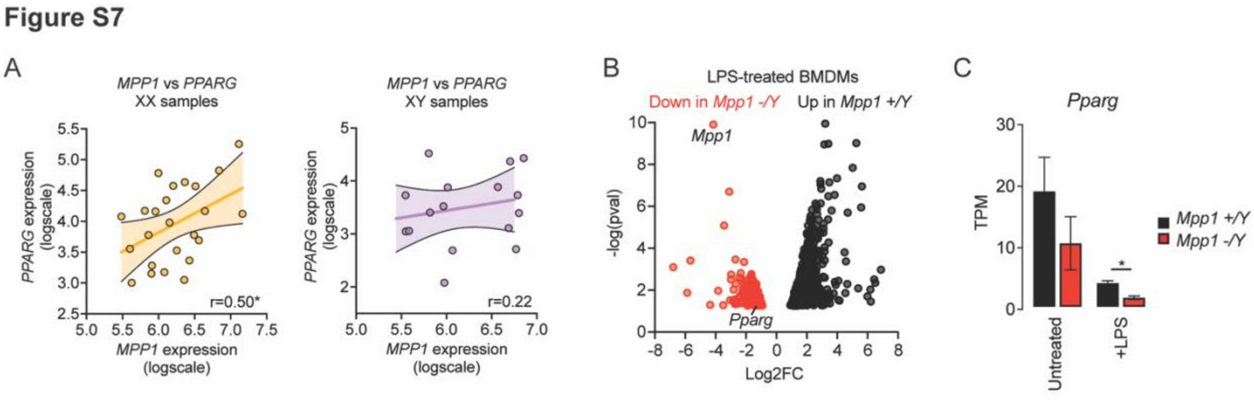

Supplementary Figure 7.

A. Correlation of *MPP1* and *PPARG* XX and XY human control microglia and TAM-MG samples.

B. Volcano plots of differentially expressed genes in *Mpp1 -/Y* vs *Mpp1 +/Y* LPS-treated BMDMs.

C. *Pparg* expression in *Mpp1 -/Y* and *Mpp1 +/Y*, LPS-treated and untreated BMDMs.

bioRxiv preprint doi: https://doi.org/10.1101/2024.06.06.597433; this version posted June 6, 2024. The copyright holder for this preprint (which was not certified by peer review) is the author/funder, who has granted bioRxiv a license to display the preprint in perpetuity. It is made available under aCC-BY 4.0 International license.

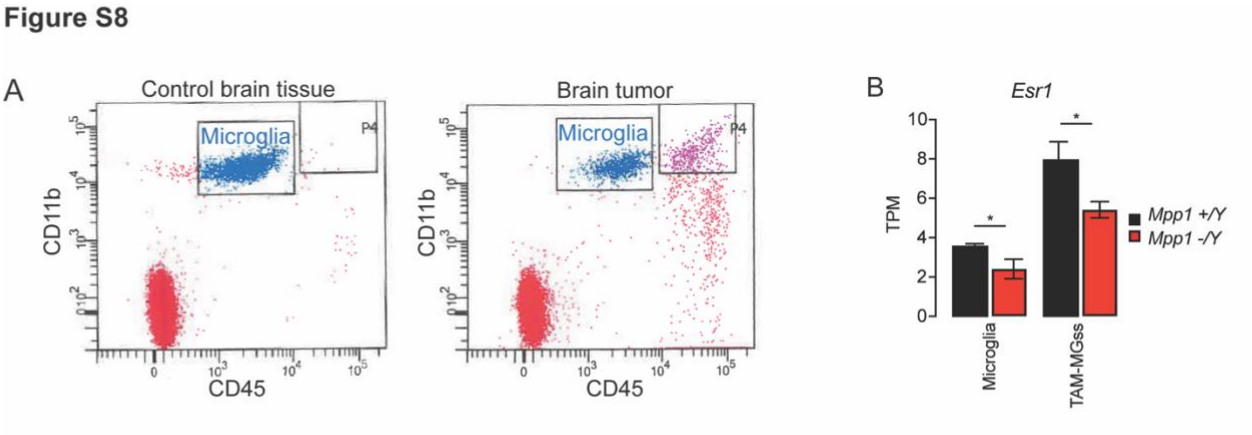

Supplementary Figure 8.

A. FACS-gating strategy for mouse control microglia and TAM-MGs.

B. *Esr1* expression in *Mpp1 -/Y* and *Mpp1 +/Y* control microglia and TAM-MGs.
